# Caching Joint Shortcut Routing to Improve Quality of Service for Information-Centric Networking

**DOI:** 10.3390/s18061750

**Published:** 2018-05-29

**Authors:** Baixiang Huang, Anfeng Liu, Chengyuan Zhang, Naixue Xiong, Zhiwen Zeng, Zhiping Cai

**Affiliations:** 1 School of Information Science and Engineering, Central South University, Changsha 410083, China; bxhuang@csu.edu.cn (B.H.); afengliu@mail.csu.edu.cn (A.L.); zengzhiwen@mail.csu.edu.cn (Z.Z.); 2 The State Key Laboratory of Industrial Control Technology, Zhejiang University, Hangzhou 310027, China; 3 Department of Mathematics and Computer Science, Northeastern State University, Tahlequah, OK 74464, USA; xiongnaixue@gmail.com; 4 Department of Network Engineering, School of Computer, National University of Defense Technology, Changsha 410073, China; zpcai@nudt.edu.cn

**Keywords:** Information-Centric Networking, routing shortcut, cooperative pre-caching, Quality of Service

## Abstract

Hundreds of thousands of ubiquitous sensing (US) devices have provided an enormous number of data for Information-Centric Networking (ICN), which is an emerging network architecture that has the potential to solve a great variety of issues faced by the traditional network. A Caching Joint Shortcut Routing (CJSR) scheme is proposed in this paper to improve the Quality of service (QoS) for ICN. The CJSR scheme mainly has two innovations which are different from other in-network caching schemes: (1) Two routing shortcuts are set up to reduce the length of routing paths. Because of some inconvenient transmission processes, the routing paths of previous schemes are prolonged, and users can only request data from Data Centers (DCs) until the data have been uploaded from Data Producers (DPs) to DCs. Hence, the first kind of shortcut is built from DPs to users directly. This shortcut could release the burden of whole network and reduce delay. Moreover, in the second shortcut routing method, a Content Router (CR) which could yield shorter length of uploading routing path from DPs to DCs is chosen, and then data packets are uploaded through this chosen CR. In this method, the uploading path shares some segments with the pre-caching path, thus the overall length of routing paths is reduced. (2) The second innovation of the CJSR scheme is that a cooperative pre-caching mechanism is proposed so that QoS could have a further increase. Besides being used in downloading routing, the pre-caching mechanism can also be used when data packets are uploaded towards DCs. Combining uploading and downloading pre-caching, the cooperative pre-caching mechanism exhibits high performance in different situations. Furthermore, to address the scarcity of storage size, an algorithm that could make use of storage from idle CRs is proposed. After comparing the proposed scheme with five existing schemes via simulations, experiments results reveal that the CJSR scheme could reduce the total number of processed interest packets by 54.8%, enhance the cache hits of each CR and reduce the number of total hop counts by 51.6% and cut down the length of routing path for users to obtain their interested data by 28.6–85.7% compared with the traditional NDN scheme. Moreover, the length of uploading routing path could be decreased by 8.3–33.3%.

## 1. Introduction

Rapid advances in the manufacture of sensing devices such as smartphones [1,2,3,4], iPad or other sensing nodes [5,6,7,8,9] have expanded the range of ubiquitous sensing applications in the Internet-of-Things (IoT) [3,9,10,11,12,13], such as monitoring and gathering information from public infrastructure [14,15,16,17,18], natural disaster relief [19,20], healthcare [21,22], smart homes [23,24], and industries [11,16,17,25,26]. The enormous number of ubiquitous sensing (US) devices provides an immense number of data (in this paper, the term “data” refers to videos, sounds, pictures or other forms of information that could be collected by sensing devices) for Information-Centric Networking (ICN) [27], which is an emerging network architecture with the potential to solve a great variety of issues faced by traditional network [27]. In addition, those applications formed the so-called Sensor-Cloud Network (SCN) [28], which is an emerging network architecture of ICN [27]. In such a network, many sensing devices which are deployed on the network edge are used for collecting data [2,4,29,30,31,32,33]. Then, sensing devices send data to cloud [28,34], where Data Centers (DCs) are located. Finally, users get requested data from DCs. Ubiquitous sensing devices, including smartphones, cameras and iPads have been changing the ways we interact and communicate dramatically, which lead to remarkable changes in the existing network [35,36,37,38,39]. On the one hand, it is the enormous number of these ubiquitous sensing devices that make it possible for data to be collected and sensed in such a huge scale [25]. According to the statistics [1,3], the total number of sensing devices (smartphones and industrial sensing devices) that are connected to the IoT network had reached 90 billion in 2011, which outnumbered the global population by that year. Moreover, this number is expected to ascend by 240 billion in the immediate future [1,3]. On the other hand, the growth of streaming traffic has also shown an exponential trend under pervasive sensing [1,3]. Due to these powerful sensing devices, people could sense videos, sounds and photos anytime and connect their devices to the Internet using various access channels. The convenience brought by sensing devices make it possible for hot events, regional conflicts and political issues to be spread worldwide almost simultaneously. Because of the colossal number of sensing devices around the world, the number of data produced by the IoT has been rising exponentially [1,3,40,41]. Reports from Cisco illustrate that the network flow of streaming produced by the IoT had accounted for 69% of the total flow in 2014, which had increased to 30-fold of the total network flow in 2000. This number is expected to grow more dramatically in the future [1,3].

The conventional Cloud Computing model [1,25,28,34] and the Sensor-Cloud Network [28,34] have to face considerable challenges brought by the rapid development of ubiquitous sensing [42,43,44]. In a traditional model, all the sensed data collected by sensing devices have to be uploaded to DCs which are located in the cloud, then it is DCs’ job to reply every incoming interest packet with a data packet with a specified size and name same as in that interest packet. Nevertheless, this model has a few disadvantages: (1) It causes heavy traffic load for DCs and backbone network because the network center where DCs are located is the core of data computing and downloading [45,46]. On the one hand, DCs have the burden of computation and data downloading. On the other hand, tons of streaming data collected from sensing devices are uploaded to DCs, which results in significant traffic flow between DCs and backbone network. (2) Traditional model can lead to poor Quality of Service (QoS) for users [47]. To obtain the first-hand information such as emergency events, public hot issues, or interesting news, users have to go through some prolonged routing processes. Firstly, newly sensed data are uploaded towards DCs; secondly, users request their interested data from DCs; and, finally, demanded data would be transferred from DCs to users. Therefore, these interacting processes result in long delay for a user to get requested data and aggravate the burden of network, thereafter it is the time lag of data transmission that causes poor QoS. Nowadays, located on the edge of the network, many devices have high storage capacity but their potential has not been fully exploited. State-of-art schemes such as Fog Computing [48] and Edge Computing network [24,45] are trying to make use of the resources on the edge of network. However, if the fundamental processes remain unchanged, the abundant computation and storage resources cannot be fully exploited.

Some researchers noticed the problems faced by the Sensor-Cloud Network. Among various proposed schemes, caching is widely used to address many of existing issues [24,35]. In those caching schemes, the major idea is that, when a user requests data, the requested data would be cached in routers along the transmission path of that data. When other users request the same data, they can directly fetch the data from routers, and the traffic burden of delivering data would hence be released and QoS would be ameliorated [35,47].

Nevertheless, the majority of past research mainly concentrates on downloading routing from DCs to users. However, besides the downloading process between users and DCs, there are a few other routing processes in SCN. In the initial place, all data are collected from sensing devices on the network edge, and users could get access to their interested data after the data have been uploaded to DCs. Therefore, there are three main processes from when data are produced to when the data are obtained by users: (1) Sensing devices collect and upload data to DCs. (2) Users request specific data from DCs. (3) DCs send the requested data towards users. These three processes are intimately correlated; hence, the distribution and transmission of data would be influenced if any of them is ineffective. The goals of previous caching schemes are to reduce data traffic or delay. Although those works are mainly based on the third process and pay little attention to the remaining processes. In this paper, we believed that the other two processes are also critical and if all of the processes are considered and optimized together, the network performance could be further enhanced. Thus, we proposed a Caching Joint Shortcut Routing (CJSR) scheme to optimize the overall performance of the network. Compared with other works, the major innovations of this work are as follows:

(1) Two shortcut routing methods are proposed to reduce the burden of network and enhance uses’ QoS. The first shortcut routing method is to build shortcut from DPs to users. In the SCN, the sensed data collected from sensing devices has to be uploaded to DCs before it can be requested by users later. However, sensing devices and users are both located on the edge of network, which means that sensing devices (DPs) and users are close to each other. Making use of this advantage in distance, the data could be directly sent to users after the formation of the shortcut. The second shortcut routing method will choose a Content Router (CR) that could yield shorter overall length of uploading routing paths, and then data packets are uploaded through this chosen CR. In this method, the uploading path shares some segments with the pre-caching path thus the overall length of routing paths is reduced. By implementing these shortcut routing methods, the latency for users to get their interested data would decrease and a better QoS could be guaranteed.

(2) A cooperative pre-caching mechanism consisting of uploading pre-caching and downloading pre-caching is proposed so that the efficiency of pre-caching could be improved further. In the CJSR scheme, pre-caching is not only performed between DCs and users similar to past schemes, but data are also cached providently during their uploading process. In the uploading pre-caching, before data have been uploaded to DCs, they would be cached in CRs by the time users request then, which would result in better QoS because users could fetch data from nearby CRs. Besides the proposed uploading pre-caching, the CJSR scheme also provides pre-caching for downloading process so that it is more adaptive to different situations and could enhance the system performance. Moreover, this paper also proposes a space-optimize algorithm that could make use of storage of idle CRs to address the scarcity of storage capacity. Overall, the CJSR scheme changes the conventional one-way model of pre-caching and enhance the network performance with a broader perspective.

(3) The effectiveness of the CJSR scheme is evaluated through extensive simulations. Because of the two kinds of shortcuts, the CJSR scheme could reduce the overall length of routing paths, decrease delay, release transmission burden and ameliorate user’s QoS. Moreover, with cooperative pre-caching, the caching feature of ICN could be exploited so that the network traffic burden is decreased further. In addition, to utilize the caching storage of each CR in a balanced way, another pre-caching scheme that could make use of idle CRs is proposed, which could relieve the scarcity of storage space. After conducting a series of experiments, we demonstrate that, compared with the traditional NDN scheme, the CJSR scheme could reduce the total number of processed interest packets by 54.8%, enhance the cache hits of each CR and reduce the number of total hop counts by 51.6% and cut down the length of routing path for users to obtain their interested data by 28.6–85.7%. Moreover, the length of uploading routing path could be decreased by 8.3–33.3%.

The rest of this paper is organized as follows: in Section 2, related works are reviewed. The system model is described in Section 3. In Section 4, a novel Caching Joint Shortcut Routing (CJSR) scheme is presented. Section 5 presents our experimental results and comparisons with literature methods. Finally, a conclusion and future works are presented in Section 6.

## 2. Related Work

Along with far-reaching development of the Internet, the traditional TCP/IP network has shown many defects facing the rapid growth of network scale as well as an ever-increasing number of data. These problems include low network performance, poor QoS and inefficient data transmission among DPs, DCs and users. Therefore, researchers carried out many relevant studies to improve data caching mechanisms, architectures of network and routing methods [27,49,50]. As a future network architecture, Information-Centric Networking (ICN) [27,51] has brought much attention from both academia and industry. In ICN, named data become a major unit of network transmission, Named Data Networking (NDN) [27,51] is a paradigm of ICN architecture. In NDN, the IP in middle layer is replaced by the named data. The transmission of data follows a “publish-request-respond” model which directly uses the named data in routing, thus the efficiency from point to multi-point transmission is improved.

Majeed et al. [35] and Chiocchetti et al. [51] indicated that caching strategy is a crucial factor to enhance network performance for NDN. In the initial place, past research proposed a Cache Everything Everywhere (CE2) scheme, of which the cached contents (the words “content”, “data packet” and “Data” are used interchangeably in this paper) tend to be homogenized and thus result in a large amount of redundancy. Due to the lack of consideration on the heterogeneous feature of contents, efficient caching of contents cannot be achieved in the CE2 scheme. Therefore, to exploit the potential of caching, designing an effective caching algorithm becomes a prime goal for NDN.

Many researchers brought up various caching methods. The earliest caching scheme of NDN is LCE (Leave Copy Everywhere) Caching Scheme, in which an interest packet finds outgoing faces by looking up entries in the Forwarding Information Base (FIB), then an entry about the request content is created in the Pending Interest Table (PIT). The corresponding data packet will be sent back along the same path that the interest packet had travelled, and then it is cached by each CR along this path [52]. However, the storage capacity of CRs is limited and copying same content everywhere causes excessive redundancy. Caching mechanism in the LCE would not only waste valuable storage space, but also render low efficiency due to the frequent replacement of contents in CRs. Therefore, the LCE caching scheme is easy to deploy but inefficient.

Earlier, Laoutaris et al. proposed the LCD (Leave Copy Down) [17], MCD (Move Copy Down) [53] and Prob (copy with probability) [53] schemes. In the LCD scheme, data are only cached in next vertex of the vertex where a cache hit happens. In the MCD scheme, when a cache hit happens, the same data would be deleted in this vertex, and then those data are cached in next vertex, similar to the LCD scheme. In the Prob scheme, each CR caches a data packet based on a predefined probability p (0<p<1). These three schemes can reduce the redundancy of cached data in the whole network to some extent. In addition, the LCD and the MCD scheme also consider the frequency of requested data. When data are requested frequently, they would be cached closer to users. Nevertheless, in these two schemes, the vertices that are close to servers have higher burden than the other vertices and the potential of those underused vertices are not fully exploited.

Chai et al. [54] proposed the Betw scheme, in which the importance of a vertex is judged by calculating its betweenness (higher betweenness denotes higher importance). In the process of forwarding data to users, the data would be cached in the vertex with the highest betweenness. In this scheme, each vertex has to compute its own betweenness; higher betweenness means that this vertex is more significant, thus caching data in such a vertex render a higher probability for users to get their interested data, and the network performance is enhanced. However, the Betw scheme would put higher caching pressure on vertices with high betweenness, and the storage capacity of low-betweenness vertices is not efficiently used. Moreover, data replacement in high-betweenness vertices is frequent, which means popular data are also likely to be replaced very often.

Psaras and Chai et al. [55] proposed the Prob Cache scheme. In this scheme, after sending an interest packet, a probability value would be calculated using the storage capacity of a CR on the path that the interest packet had just travelled and the distance between this CR and users. Then, this number would be referred as a criterion on caching data packets. The Prob scheme is also used widely by later research. Overall, it is a scheme that, after evaluating the importance of each vertex, cache data according to the importance of vertices. This scheme reduces redundancy of data packets to some extent, improves efficiency on using storage capacity and ameliorate the network performance. However, popularity of data is not considered in this scheme, and all the data are treated equally. As a result, the popular contents are cached inefficiently.

Ming et al. [56] proposed the age of cache, which is calculated by using popularity of a content and the position of the vertex that stores this content. This work also combined age with other factors to enhance the performance of schemes that simply replace the old content with a new one. Cho et al. [57] proposed a collaborative WAVE scheme that based on the popularity of content packets, the number of cache on a content packet grows exponentially according to the number of request on this content. In addition, Zhang et al. [58] made a comparison on existing caching schemes, indicating that there was a lack of consideration on the distribution attributes of content requests in the design of those caching algorithms. They also gave an inspiration on possible future research directions.

Cache-based technologies is of great importance for NDN, however the development of network has risen new challenges for these technologies. In fact, existing caching schemes assume that data have always been uploaded to DCs by the time it is requested by a user, and the above-discussed schemes are all based on this assumption. However, such an assumption is only applicable for network ten years ago; the concept of = Cloud Computing has since been proposed. The core idea of the Cloud Computing is to deploy hardware resources that have powerful computation ability and high storage capacity in network center; it is similar to the structure of fat server that can undertake a large number of complicated computation and storage. On the other hand, a terminal is rather simple, which gets the result of computation after sending request to the cloud. In this way, the model of Cloud Computing hides the physical difference of devices and provide distributed services. Nevertheless, with the development of IoT, the number of devices that are connected to the Internet grows substantially [1,3,59]; the scale and range of producing data and sharing data also increase exponentially. Because all the data produced by numerous devices must be sent to network center (Cloud platforms or DCs) for computing and processing, the overall burden of the network center is significant, delay is high and QoS is poor. On the one hand, devices in the network center are overloaded and network is often congested. On the other hand, computation and storage resources of many devices on the edge are underused. Thus, computational frameworks such as Fog Computing and Edge Computing are proposed. Their main idea is to shift the work of computing from network center to edge to exploit the potential of computation and storage resources. Under that model, users’ request would be satisfied in a local network. The congestion and latency caused by long-distance transmission between users and DCs could be decreased. However, the research of distributed network architectures is still in early stage of exploration.

Because the emerging network architectures are immature, the original network architecture is still widely used, but its caching mechanisms are inefficient. Specifically, those caching schemes are only applicable during routing between DCs and users. In other words, it could only solve partial problem of down data flow from network center to network edge. Performance of the process of uploading immense data from sensing devices to DCs has not been improved by past schemes, not to mention, more broadly, trying to optimize those processes together. Nowadays, main pressure on the network is caused by the up data flow, or the data flow from edge to edge, which has to be relayed by network center (for instance, a colossal number of data produced by instant messaging such as QQ and WeChat must be relayed by network center). In conclusion, the CJSR scheme in this paper could be a possible solution based on such a background; this scheme considers the attributes of present network and aims to solve the existing problems.

## 3. System Model and Problem Statement

### 3.1. Network Model

**Figure 1:**
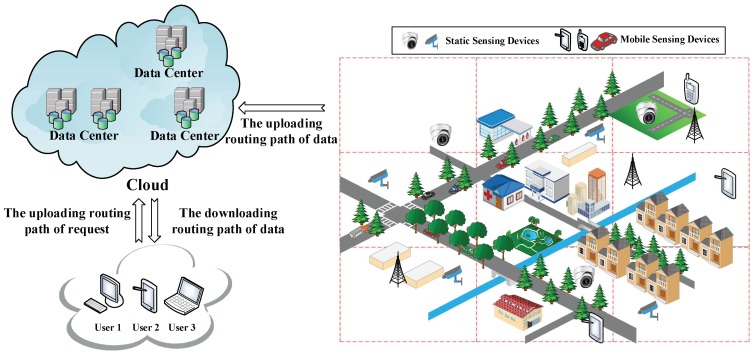
The architecture of the network.

As shown in Figure 1, the network model used in this paper is Sensor-Cloud Network (SCN), which is an emerging network model in ICN. In such a network, there are three major components:

(1) Ubiquitous sensing devices. Broadly distributed sensing devices are producers of data. The information foundation of ICN is based on a colossal number of data collected and sensed by these devices and the number of these devices are numerous. Located on the network edge, they are important parts of the Internet of Things (IoT). These sensing devices are made up of mobile sensing devices such as portable devices (smartphones, iPad and cameras) and more powerful devices carried by vehicles, ships and unmanned aerial vehicles. There are also static sensing devices including surveillance cameras and industrial sensors. Data can be sensed in formats such as video, audio, image or text. For instance, emergency events or breaking news are usually collected by sensing devices of nearby people in forms of videos, audios and images. Following that, the collected content is uploaded to DCs for storage. Ubiquitous sensing devices are shown in the left part of Figure 1.

(2) Data Centers (DCs). DCs are similar to publishers in Named Data Networking (NDN), which provide all the requested data to users and have the most comprehensive storage of all kinds of data. Rather than focus on the transmission of stored video streaming similar to other research on NDN, the data of SCN in this paper have semi-real-time attribute. When an emergency event or breaking news happens, users want to obtain relevant videos, audios and images as soon as possible. Lagged data transmission would result in loss of interest; these data also become out-of-date as time goes by. For instance, during a traffic congestion, drivers in a congestion area send the current traffic situation to a DC, other users could get this information and use it to schedule their routes. If the obtained data are outdated, users (especially those who plan to drive towards this road) would make wrong decisions. One possible situation is that the outdated information may lead users to drive through a longer road because they thought the shorter road is congested. As a result, this longer road would cost more time and gasoline. Users could also be misled to a congested road by outdated information which reports that this road is unobstructed. Overall, this information would give users a poor QoS. Besides the example we give above, similar problems also happen on other applications and could cause users to make wrong decisions. In this paper, DCs are different from those in traditional ICN, in which it is assumed that all data have been uploaded whenever users request them. Traditionally, data are usually obtained by physical methods such as installing storage hardware (hard disks, CDs or tape recorders). Those data are mainly multi-media streaming with low real-time request. Thus, data producers in traditional ICN are usually omitted and only the interaction process between DCs (publishers) and users is considered. However, in this paper, we aim to improve performance on processing semi-real-time data, and have considered a situation that the requested data have not been uploaded completely. Besides stored videos used in traditional ICN, we focus on real-time videos collected from sensing devices around locations where breaking news occurred, and collected video streaming is often short (approximately lasts between a few seconds and ten minutes). Such real-time data would provide users better QoS if they could be delivered more swiftly; vice versa, outdated data have less value and QoS would be declined under their influence.

(3) Users. In this paper, users are equivalent to data requesters, such as drivers in the aforementioned example, or people who are interested in breaking news or a hot event. Due to the powerful functions of smartphones, more and more users obtain data through smartphones or iPad rather than personal computers. Thus, smartphones are not only producers (Data Producers of DCs) which sense and collect data, but also data consumers. It is also noticeable that users and DPs (sensing devices) are both located on the network edge.

Besides the three components discussed above, the network model contains the following interaction processes:

(1) Routing process of requesting data from DCs. Although users and DPs are both located on the network edge, prefixes of sensing devices are unknown to users, but prefixes of DCs are public to the whole network. Therefore, a user would firstly request data from DCs as they usually do in conventional SCN. This routing path is built between users and cloud; we refer to it as routing path of request. To build the first kind of shortcut that enables users to directly send requests to sensing devices rather than DCs, a DP’s prefix would be recorded as soon as a DC receives uploaded data. Hence, with the prefix recorded, the DC could reply the information of the DP to the user if the requested data have not been uploaded yet. Then, the user could directly request data from the DP. Otherwise, the DC would send corresponding data to the user following the traditional procedures.

(2) Routing process of transmitting data from DCs to users. When DCs receive requests from users, they send corresponding data to users if the requested data are stored. This routing path is the transmission path of data between DCs and users, thus this path is referred as downloading routing path of data.

(3) Routing process of uploading data. While sensing devices are collecting data, they upload collected data to DCs at the same time without being requested by DCs. The data collection process in the network model which our work used is similar to crowdsourcing network or participatory network [14,15,16,17,18,19]. Therefore, data packets are uploaded without being requested by the cloud. The routing path in this process is referred to as the routing path of uploading data. This routing path is usually directly built among DCs and DPs. However, using two shortcut routing methods discussed in Section 4, this path would adjust itself to yield shorter overall routing paths.

Next, we give standardized definition of the network model shown in Figure 1. The network can be defined as an undirected graph *G* = (ξ, *E*), where ξ is a set of vertices or nodes representing content routers, data requesters (DRs), data producers (DPs) and the data centers (DCs). Usually, DRs, DPs and DCs are located on the terminal of a routing path while CRs are intermediary. *E* is the set of undirected edges modeling communication links between pairs of vertices.

(1) Suppose that ξ = $\left\{\xi_{i1},\xi_{i2},\ldots ,\;\xi_{in}\right\}$ is a set of intermediary content routers (CRs) on a routing path (start with a requester and end at a DC or a DP). The CRs can cache the data in their cache storage. A vertex$\;\xi_{i}$ has a limited cache storage capacity $C_{\xi_{i}}$, measured in bytes.

(2) Suppose that $R=\left\{r_{1},r_{2},\ldots ,\;r_{m}\right\}$ is the set of data requesters (DRs), i.e., users. The data requesters request data and if the data have been cached among CRs, the hop count for users to obtain these data would be reduced. Users could obtain data from a CR with these data cached, thus the QoS is ameliorated. Similar to a previous research [35], we assume that data requesters are connected to network through a single CR. We denote the vertex (CR) connected to requester $r_{i}$ as $\xi_{r_{i}}$.

(3) Suppose that $S=\left\{p_{1},p_{2},\ldots ,\;p_{k}\right\}$ is the set of data producers (DPs). The data producers are sensing devices that sense and collect data. Sensing devices provide data to users or DCs through CRs. Different with other research, data can also be cached during the transmission from DPs to DRs in our work. We denote the vertex (CR) connected to data producer $S_{i}$ as $\xi_{p_{i}}$.

(4) Suppose that $D=\left\{D_{1},D_{2},\ldots ,\;D_{N}\right\}$ is the set of data centers (DCs). Data Centers have powerful storage and computation capacity and their addresses are public. Besides the data that have not been uploaded, they have stored all the data that may be requested by users. We denote the vertex (CR) connected to data center $D_{i}$ as $\xi_{D_{i}}$.

(5) Let $S_{n}$ = {$S_{1},S_{2},\ldots ,S_{m},\ldots ,\;S_{n}$} be a set of data packets. For a requested chunk $S_{m}$ from $S_{n}$, we define L as the fixed size of each data packet. $A_{\xi_{i,r_{j}}}^{m}$ denotes the frequency of interest packets for $S_{m}$ at node $\xi_{i}\in V$ from user $r_{j}$.

(6) Suppose that $\mathbb{P}=\left\{P_{1},P_{2},\ldots ,P_{i},\ldots ,\;P_{M}\right\}$ is a set of routing path. $P_{i}=\left\{\xi_{i1},\xi_{i2},\ldots ,\;\xi_{in}\right\}$, in which $\xi_{i1}$ is the first vertex on this routing path. There are a few possible scenarios: (a) When users start to request data through the uploading routing path, $\xi_{i1}$ is a user ($\xi_{r_{i}}$), while $\xi_{in}$ could be a CR, a DC or a DP, if the request is satisfied by a CR in the routing path, $\xi_{in}$ is that CR; if the request is satisfied by a DC, $\xi_{in}$ is that DC ($\xi_{D_{i}}$); if the requested data have not been uploaded yet, users will request from a DP, in that case, $\xi_{in}$ is a DP ($\xi_{p_{i}}$). To facilitate pre-caching, we make use of the transmission path of an interest packet from user to DCs or DPs, and we refer to this path as $P_{cache}$. $P_{cache}=\left\{\xi_{i1}\left(\xi_{r_{i}}\right),\xi_{i2},\ldots ,\;\xi_{ij}\left(\xi_{p_{i}}\;or\;\xi_{D_{i}}\right)\right\}$. We discuss the details of its construction in Section 4.1. (b) During the transmission of data, $\xi_{i1}$ could be a CR or a DC while $\xi_{in}$ is the user who issues this request ($\xi_{r_{i}}$). (c) For the uploading routing path of data, $\xi_{i1}$ is a DP, and $\xi_{in}$ could be a user, CR or DC. If $\xi_{in}$ is a user, it denotes that data are forwarded from a DP to a user; if $\xi_{in}$ is a CR, this routing path starts from a DP to a CR; and, if $\xi_{in}$ is a DC, it is a between a DP and a DC. Thus, the first and the last vertex on routing path $P_{i}=\left\{\xi_{i1},\xi_{i2},\ldots ,\;\xi_{in}\right\}$ is usually $\xi_{r_{i}}$, $\xi_{D_{i}}$ or $\xi_{p_{i}}$.

For the convenience of readers, Table 1 summarizes the notations used in this paper.

### 3.2. Problem Statement

The optimization objectives of the CJSR scheme are different from past schemes that merely focus on performing pre-caching from DCs to users. For those past schemes, their objective is to minimize the total number of hop counts for users to obtain data. This paper gives a more comprehensive consideration by taking into account of three interaction processes that we discuss in Section 3.1. Hence, the CJSR scheme is more practical for SCN. The optimization objectives in this paper include:

(1) Minimization of the total hop counts for users to obtain data. Assume that the path for users to obtain data is the same as previous definitions: $\mathbb{P}=\left\{P_{1},P_{2},\ldots ,P_{i},\ldots ,\;P_{M}\right\}$ and $P_{i}=\left\{\xi_{i1},\xi_{i2},\ldots ,\;\xi_{in}\right\}$. Then, the length of the ith routing path is denoted as$\;H_{i}$ = $\left|P_{i}\right|$. The first objective in this paper is to minimize the hop counts that users obtain their interested data, as demonstrated by Equation (1).

(1) $$\;\min\left(ℍ\right)=\min\left(\underset{1\leq i\leq M}{\sum }\left|P_{i}\right|\right)$$

(2) Minimization of delay. The optimization objective of past schemes is similar to Equation (1). Besides, we have more objectives in this paper. The delay refers to the whole time interval from when data are requested to when they are finally received by users, and reflects the timeliness for a user to receive data. Obviously, less delay would lead to better QoS. Assume the time that a data chunk $C_{i,j}$ is produced is $G_{i,j}$, the time when it is received by user k is$\;J_{i,j}^{k}$. Thus, the delay of user k requests for data chunk $C_{i,j}$ is $\Gamma_{i,j}^{k}=J_{i,j}^{k}-G_{i,j}$. Thus, the second optimization objective is Equation (2)

(2) $$\min\left(\Gamma \right)=\min\left(\overset{1\leq k\leq m}{\underset{1\leq i\leq m,1\leq j\leq 𝕞}{\sum }}\Gamma_{i,j}^{k}\right)$$

(3) Minimization of network burden. The burden of network could be measured by the number of packets forwarded by each CR. Reduce the burden is also one of the CJSR scheme’s objectives. It is assumed that the number of packets forwarded by a CR $\xi_{i}$ is $S_{i}$. The third optimization objective in this paper is Equation (3).

(3) $$\;\min\left(O\right)=\min\left(\underset{1\leq i\leq m}{\sum }S_{i}\right)$$

Obviously, the goal of the CJSR scheme is to minimize the total number of hops ℍ, minimize delay Γ and minimize network burden O. A constraint for achieving these optimization goals is: The caching storage of a CR $\xi_{i}$ at some time ($C_{\xi_{i}}$) cannot exceed its physical storage capacity $C_{\xi_{i}}$. The optimization goals in this paper can be summarized as follows.

(4) $$\{\begin{array}{c} \;\min\left(ℍ\right)=\min\left(\underset{1\leq i\leq M}{\sum }\left|P_{i}\right|\right)\\ \;\min\left(\Gamma \right)=\min\left(\overset{1\leq k\leq m}{\underset{1\leq i\leq m,1\leq j\leq 𝕞}{\sum }}\Gamma_{i,j}^{k}\right)\\ \;\min\left(O\right)=\min\left(\underset{1\leq i\leq m}{\sum }S_{i}\right)\;\\ s.t.\;\forall i\;|\;C_{\xi_{i}}\leq C_{\xi_{i}} \end{array}$$

## 4. Main Design of the CJSR Scheme

### 4.1. The Design of Data Structures

**Figure 2:**
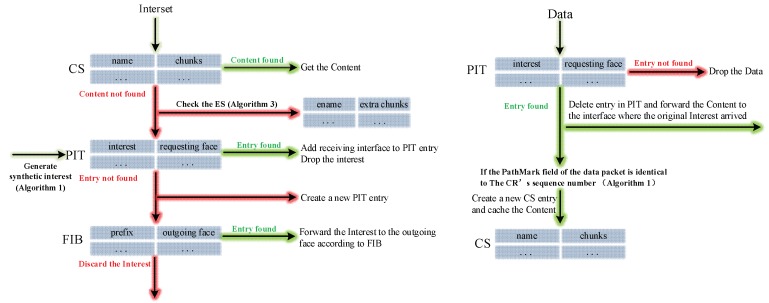
Lookup procedures of the CJSR scheme.

In this section, we discuss data structures related to the CJSR scheme. Our scheme is an improvement based on previous in-network caching schemes and we aim to improve caching mechanisms and reduce the length of routing paths. Firstly, three classic data structures including the Content Store (CS), the Pending Interest Table (PIT) and the Forwarding Information Base (FIB) are used and the lookup procedures are depicted in Figure 2. Other data structures used in the CJSR scheme include: (1) interest packet, which is used by users to request data and to construct a fixed path to facilitate pre-caching; (2) data packet, the function of which is to carry data and relevant information about data (like its destination); (3) Extra Storage (ES), which is a table sharing a similar structure to the Content Store. The ES is used in Algorithm 3 for an idle CR to help store data packets.

**Figure 3:**
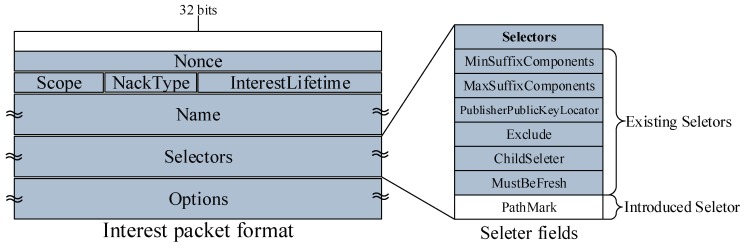
Interest packet format in the CJSR scheme.

**Figure 4:**
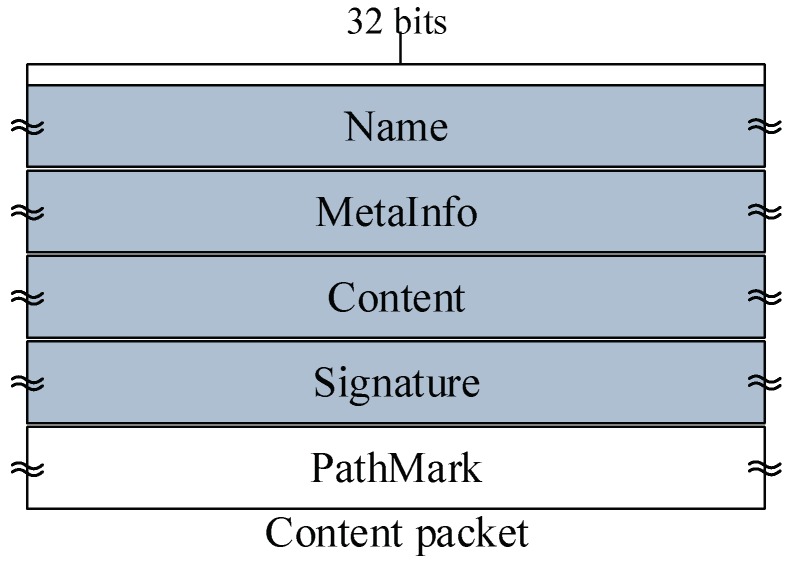
Data packet format in the CJSR scheme.

**Figure 5:**
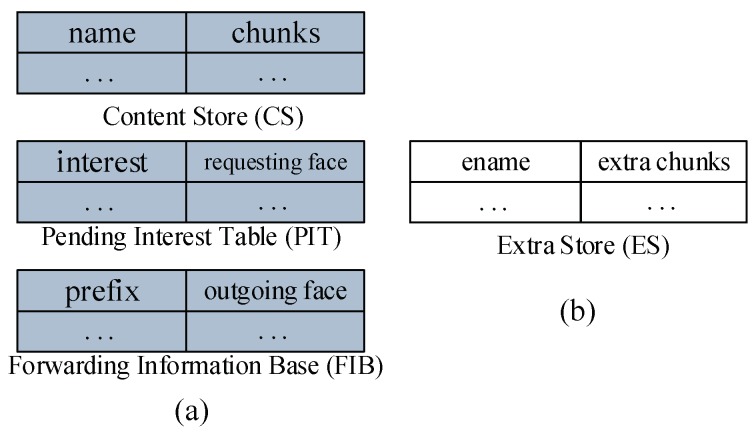
(**a**) NDN data structures; and (**b**) introduced data structure.

(1) Interest packet format. The structure of an interest packet is demonstrated in Figure 3 (the tilde marks represent that the length of this field is variable). Besides Name and Nonce, Selectors are optional elements that further qualify Data that may match the Interest. They are used for discovering and selecting the data that match best to what the application wants, and this is the place where the PathMark element is added. The aim of adding this element is to record a series of CRs that this interest packet had travelled. Thus, in the CJSR scheme, with the added PathMark, a DC or a DP can construct a fixed path to facilitate pre-caching. The reason for constructing such a path is to providently assign data packets into CRs in a controllable way. Traditionally, data packets would be sent back through the transmission path of interest packets. Therefore, it is reasonable to use this path to assign the predicted data packets to conduct pre-caching. To enable DPs or DCs to construct this path after receiving an interest packet, CRs and users would add their sequence number to an interest packet when they forward it, so the $P_{cache}$ is recorded in the Selecters field.

(2) Data packet format. A data packet represents some arbitrary binary data held in the Content element together with its Name, some additional bits of information (MetaInfo), and a digital Signature of the other three elements. The Name is the first element since all NDN packet processing starts with the name. Signature is put at the end of the packet to ease the implementation because signature computation covers all the elements before it. To assign each data packet to its correct location in pre-caching, we add a PathMark field to data packets as it is shown in the white field of Figure 4. This field is used as the indication of a data packet’s destination. Thus, $P_{m}$ would be added to the PathMark field of a produced data packet; when a CR receive a data packet, it checks whether the $P_{m}$ is same as its own sequence number and forwards this packet to next CR along the $P_{cache}$ if the $P_{m}$ does not matched.

(3) Extra Storage (ES). Figure 5b illustrates this data structure. To address the scarcity of storage space, we introduced the ES to use idle CRs. The structure and storage capacity of the ES and the CS is similar, each “extra chunks” is mapped to an “ename”. When a CR on the $P_{cache}$ asks an idle CR to cache a data packet on its behalf, relevant information is stored in the “ename” and “extra chunks” entries of the ES for future reference.

### 4.2. CJSR Scheme

The CJSR scheme is composed of Algorithms 1 and 2. The former gives detailed procedures of pre-caching, which roughly contains two steps. Step 1 describes how to process an interest packet and the assignment of data packets. Step 2 is the transmission process of a data packet. Algorithm 2 is the overall algorithm of the CJSR scheme, and gives the pseudo-code of the whole process of the cooperative pre-caching, which consists of uploading pre-caching and downloading pre-caching. In Section 4.3, we discuss a further attempt to address the scarcity of storage capacity.

The CJSR scheme could cache semi-real-time data in CRs intelligently by using the cooperative pre-caching. Moreover, with two shortcut routing methods, the length of routing path for users to obtain data is noticeably decreased. In the CJSR scheme, DPs not only collect and provide data, but also assign data towards users proactively. During the whole process, if cooperative pre-caching is conducted successfully, users could fetch their interested data from nearby CRs rather than DCs. That is to say, interest packets could be handled by CRs that have the requested data cached, thus the burden of processing interest packets would be decreased. In addition, it is also unnecessary for data packets to travel through the whole $P_{cache}$ when they are requested by users. Therefore, the burden of forwarding data packets would also be declined, due to the decline of hop counts between CRs with requested data cached and users; latency could have a noticeable drop. Thus, Equations (1)–(3) could be improved, which provides an empirical guarantee for better QoS.

As a significant component of the CJSR scheme, the pre-caching algorithm is discussed initially. First, a user sends an interest packet $I_{m}$ requesting for $S_{m}$ (e.g., a real-time video relevant to breaking news). As $I_{m}$ are being forwarded towards DCs or DPs (these two situations are discussed in the cooperative pre-caching of Algorithm 2), each CR adds its sequence number to the PathMark of $I_{m}$. Then, the $P_{cache}$ is constructed by the time this interest packet arrives at a DC or a DP. Because the same video streaming is successive, after requesting for $S_{m}$, this user is highly likely to request the subsequent data of the same streaming. In addition, due to the extensive public concern for breaking news, other users also have high probability to request the same data. Therefore, after the formation of $P_{cache}$, pre-caching is triggered and the rest of data stored in the DC or the following data collected by the DP would be assigned to CRs along the $P_{cache}$ providently. To assign unrequested data, the synthetic interest generator (discussed in NFD developer’s guide) could be used to make entries in the PIT. In other words, from the CRs’ point of view, the remaining data look like they have been requested. Thus, by the time those data packets arrive at vertices they are assigned to, they can be cached. Due to the limited storage size of each CR, each CR can only store a proportion of the data, thus the remaining data from $S_{n}$ = {$S_{1},S_{2},\ldots ,S_{m},\ldots ,\;S_{n}$} are divided into a few arrays. The size of each array depends on the cache capacity of CRs (size of each array = $C_{\xi_{i}}$ = d data packets). In the order from $S_{m}$ to $S_{n}$, the remaining data are divided into (m − n)/d arrays. The first array which has the largest probability to be requested later is cached in the nearest CR ($\xi_{r_{i}}$) next to a user ($r_{i}$). Following that, each of the remaining arrays is cached into each CR along the $P_{cache}$. Hence, each round of pre-caching can assign d×$N_{\xi }$ data packets and the CJSR scheme is consisted of (m − n)/d × $N_{\xi }$ round(s) of pre-caching. It is noticeable that only the interest packet requesting for the first data of each round can trigger pre-caching ($I_{m},\;I_{m+d\times N_{\xi }},\;I_{m+2\times d\times N_{\xi }}\;$ etc.), other interest packets would be processed by intermediary CRs rather than DPs or DCs if the pre-caching is conducted successfully. Nevertheless, an interest packet may not be satisfied by overlapped CRs (such situations are also considered in Scenario 2 and Scenario 4 of experiments); this interest has to be forwarded to the DP or the DC along the caching path and the data packet is sent back using LCE cache strategy. The specific algorithm of pre-caching is shown in Algorithm 1.


**Algorithm 1 Pre-caching**
1:**Step 1**: $I_{m}$ requesting for $S_{m}$ from $\;S_{n}$ = {$S_{1},S_{2},\ldots ,S_{m},\ldots ,\;S_{n}$} is issued by a user2:$I_{m}$ arrives at a DP or a DC3:Construct the $P_{cache}$ = {$\xi_{i1},\;\xi_{i2},\ldots ,\xi_{ij}$} from the PathMark element of $I_{m}$
4:Compute the number of vertices in the $P_{cache}$:$\;N_{\xi }$
5:(n–m) packets are divided into (m–n)/d arrays, each of which contains d packets6:**For** j = 1 to $N_{\xi }$ do7: **For** k = m + j × d − d to m + j × d − 18:  Produced a data packet $S_{k}$, add j to its PathMark field indicating its destination9:  Forward $S_{k}$ to $\xi_{ij}$
10: **End For**11:
**End For**
12:
**Step 2:**
*The transmission of a data packet*
$S_{k}$
13:**For** k = m to n14: **For** j = $N_{\xi }$ to 115:  $S_{k}$ arrives at $\xi_{ij}$
16:  **If**
$I_{k}$ is found in the PIT of $\xi_{ij}$
17:   **If** the $P_{m}$ of $\;S_{k}$ is identical to $\xi_{ij}$’s sequence number18:    Cache $S_{k}$
19:   **Else**20:    Forward $S_{k}$ to next vertex21:   **End if**22:  **Else**23:   Drop $\;S_{k}$
24:  **End If**25: **End For**26:
**End For**


Next, the CJSR algorithm is given below. Using the pre-caching discussed in Algorithm 1, the cooperative pre-caching is achieved by applying pre-caching in uploading and downloading process in two possible situations: (1) The requested data are stored in a DC. Then, the DC could perform downloading pre-caching between the user and itself. During this process, interest packets are forwarded using the prefix of the DC. Following that, the chunk is produced using DC’s signature. (2) The requested data have not been uploaded yet. In this case, the DC would respond the user’s request with the location of the DP that is collecting the requested data. Once the user gets the address of that DP, the request can be forwarded to the DP directly, and then the DP could perform uploading pre-caching. The user would use the DP’s prefix to request, while the produced data packets are signed by the DP. In other words, each chunk only has one name in these two situations, hence cooperative pre-caching is applicable for various situations. Another main idea of the CJSR scheme is the formation of shortcut. After acquiring the location of the DP, the user directly request data from the DP, since now the first kind of shortcut is constructed. In addition, the uploading process and pre-caching happens at the same time, and the uploading path of data packets would share some segments of $P_{cache}$. Then, data packets would find a CR that could yield shorter overall routing path to form the second kind of shortcut. Firstly, prefixes from Data Centers are used if users request data from DCs. After the formation of the first shortcut, the prefix of the Data Producer is used and interest packets are sent to the DP. By combining the cooperative pre-caching and two shortcut routing methods, the burden of all devices (especially DCs and DPs) could be decreased significantly, cache hits would ascend and latency would drop. Algorithm 2 depicts the concrete process of the CJSR scheme.


**Algorithm 2 Caching Joint Shortcut Routing**
1:$I_{m}$ requesting for $S_{m}$ from $\;S_{n}$
*=* {$S_{1},S_{2},\ldots ,S_{m},\ldots ,\;S_{n}$} is issued by a user2:$I_{m}$ arrives at a vertex3:**If** the pre-cached data in vertex $\xi_{ij}$ meets the demand after performing lookup on its Content Store4: Forward $S_{m}$ to user5:
**Else**
6: Forward $I_{k}$ to next vertex7: **If**
$I_{m}$ arrives at a DC8:  **If** the requested data have not been uploaded9:   The DC responds the location of DP to the user10:   The user forwards $I_{m}$ towards the DP11:   The DP performs algorithm 1 between the DP and the user (uploading pre-caching)12:  **Else**13:   The DC performs algorithm 1 between the DC and the user (downloading pre-caching)14: **End If**15:
**End If**


**Figure 6:**
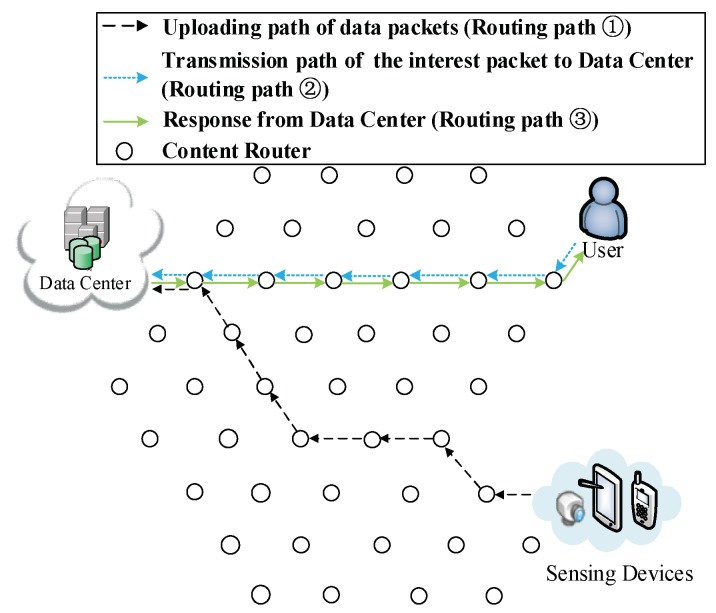
The exchange of information between the user and the DC.

**Figure 7:**
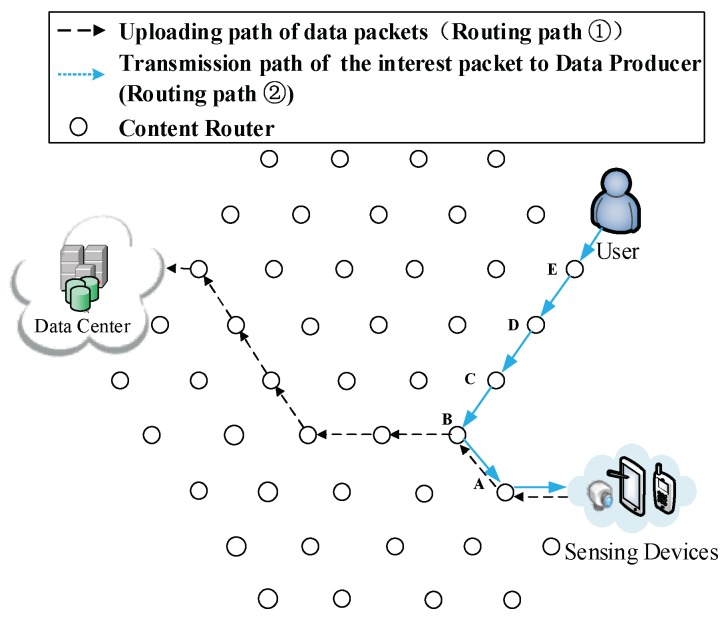
User start to request data from the DP.

**Figure 8:**
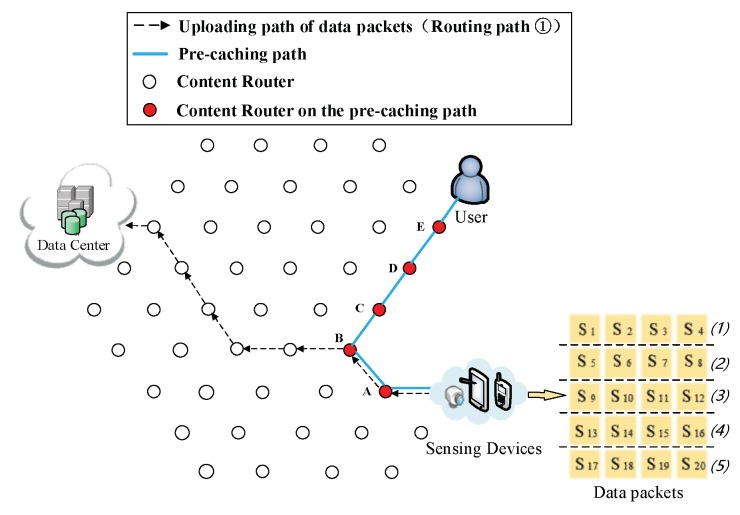
The formation of $\;P_{cache}$.

**Figure 9:**
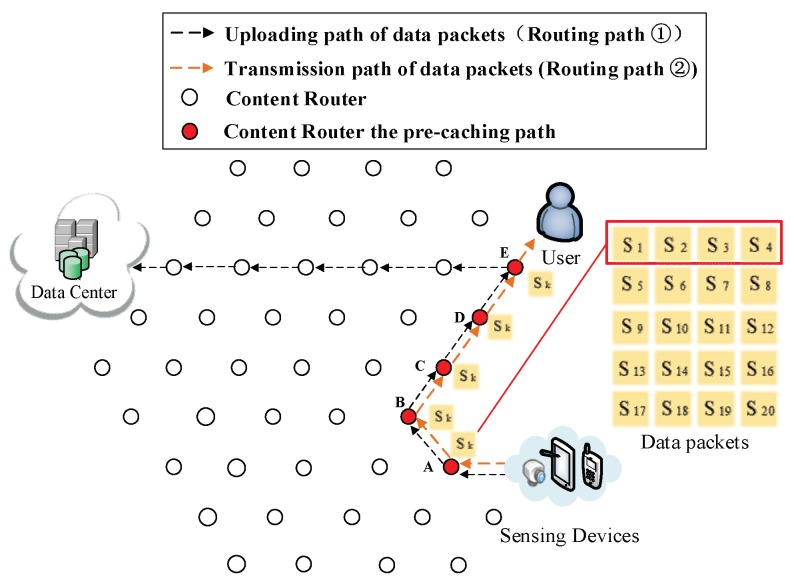
Send the first array $S_{k}$ (*k* = 1, 2, 3, 4) to Vertex E.

**Figure 10:**
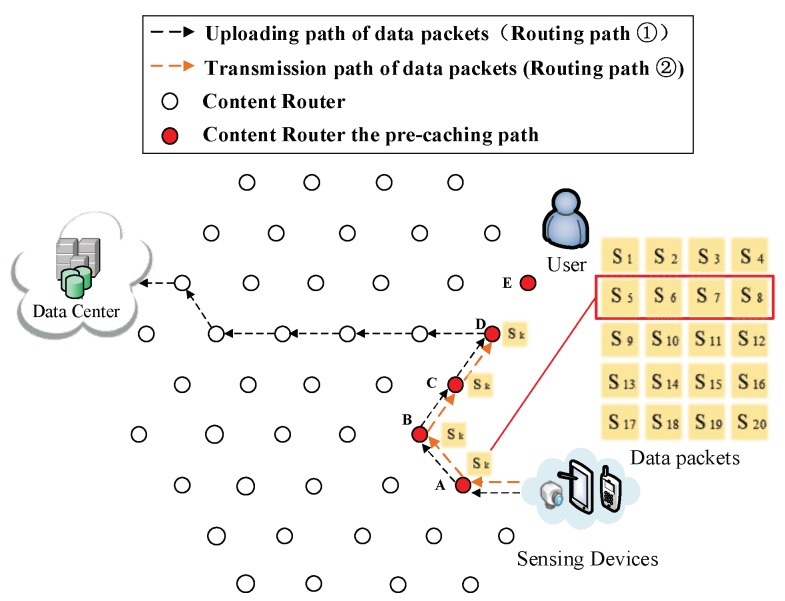
Send the second array $S_{k}$ (*k* = 5, 6, 7, 8) to Vertex D.

**Figure 11:**
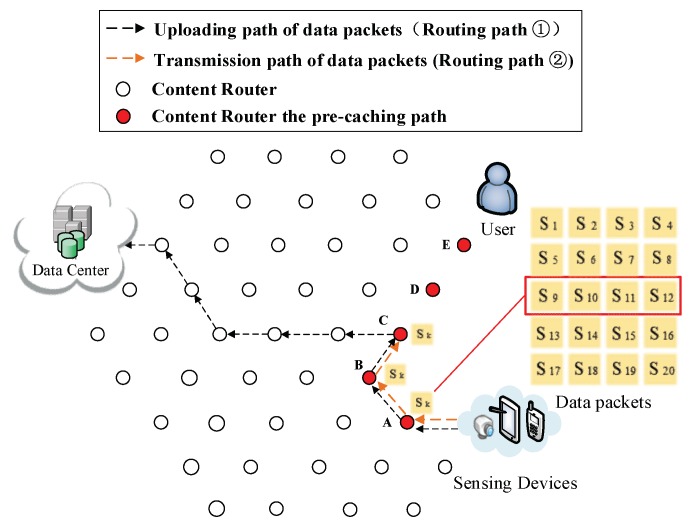
Send the third array $S_{k}$ (*k* = 9, 10, 11, 12) to Vertex C.

**Figure 12:**
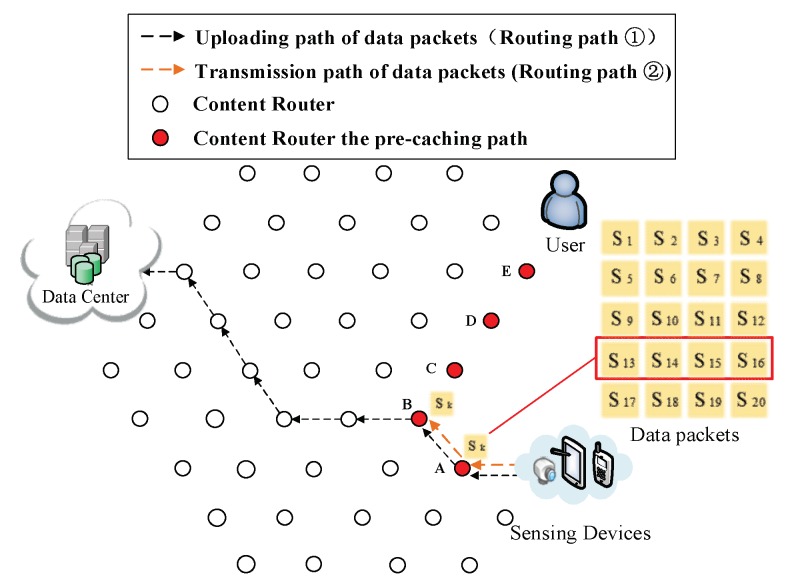
Send the fourth array $S_{k}$ (*k* = 13, 14, 15, 16) to Vertex B.

**Figure 13:**
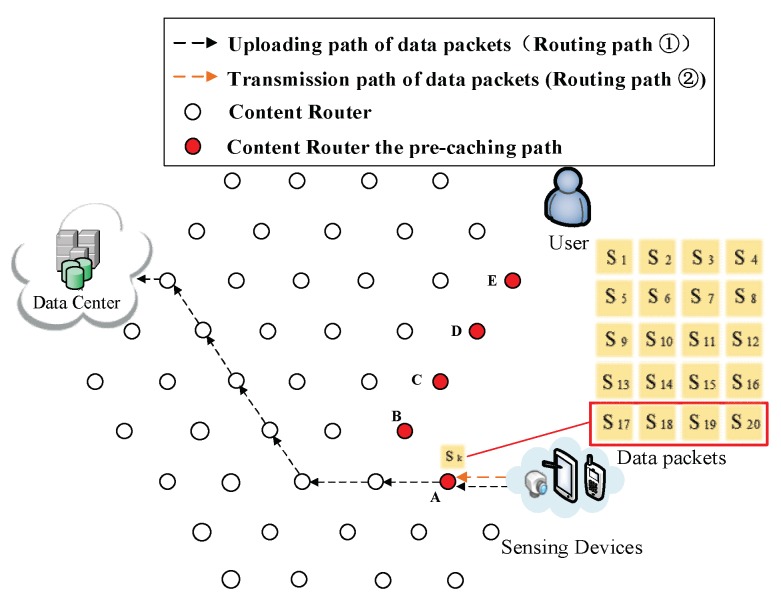
Send the fifth array $S_{k}$ (*k* = 17, 18, 19, 20) to Vertex A.

**Figure 14:**
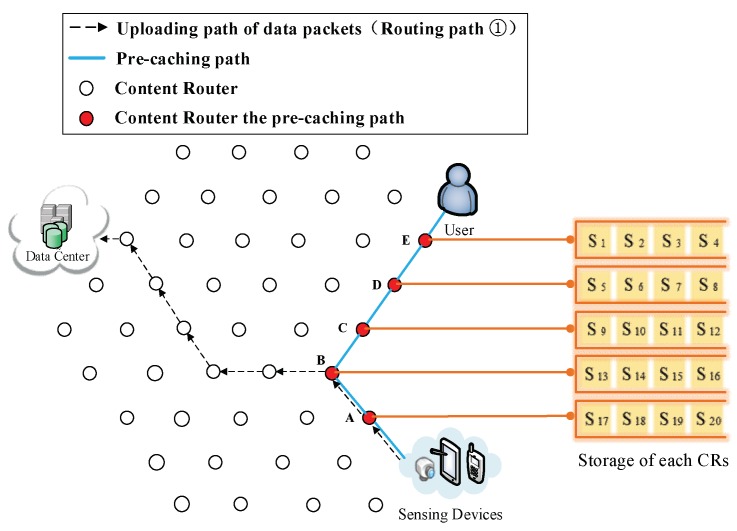
The cache storage of each CR along the $P_{cache}$.

**Figure 15:**
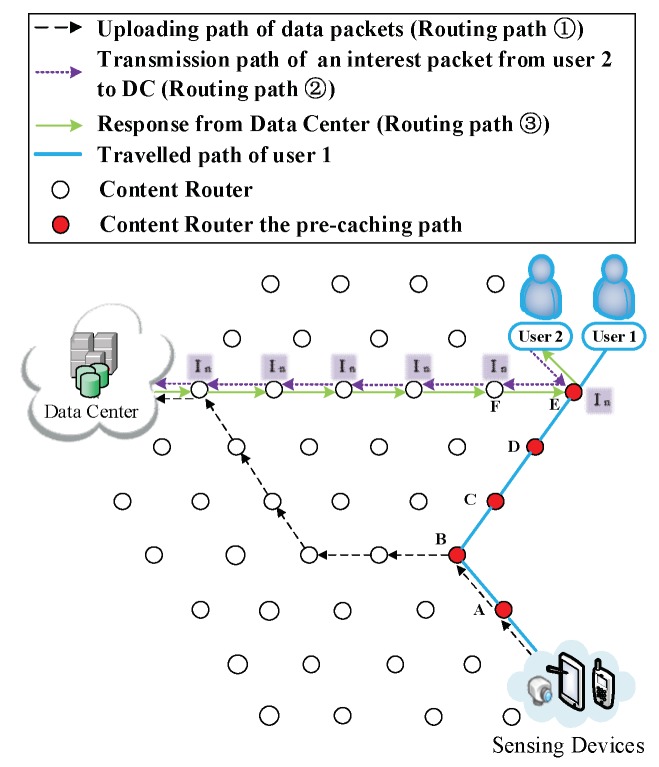
User 2 joins the network demanding different data.

**Figure 16:**
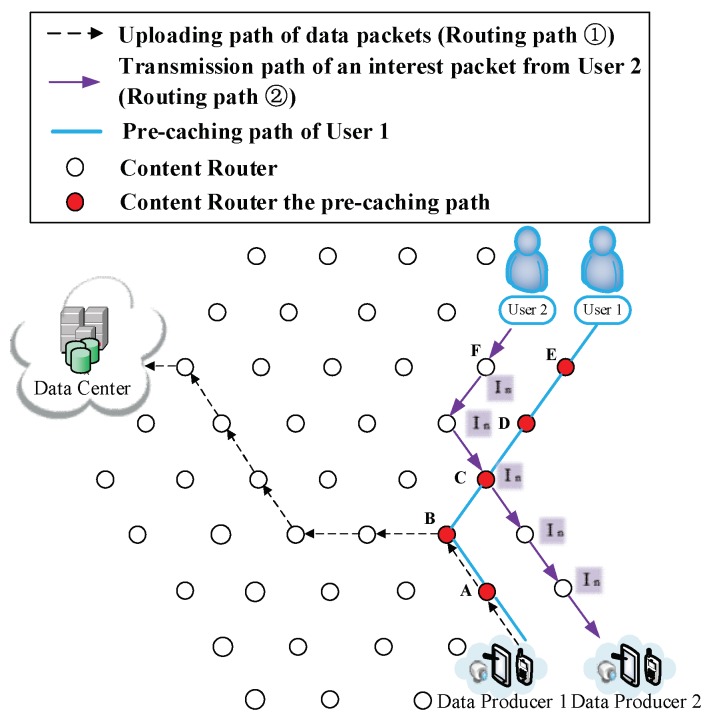
User 2 starts to request from the DP.

**Figure 17:**
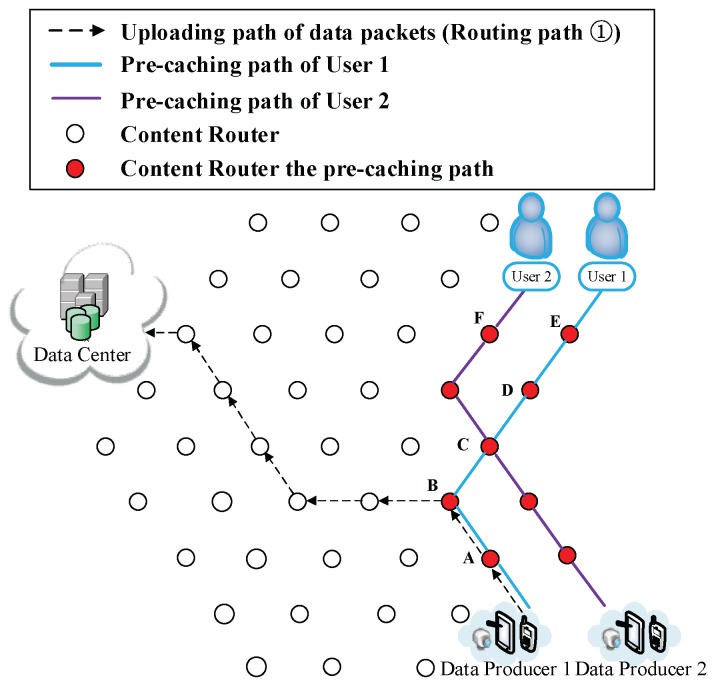
Two users build up their own caching path.

**Figure 18:**
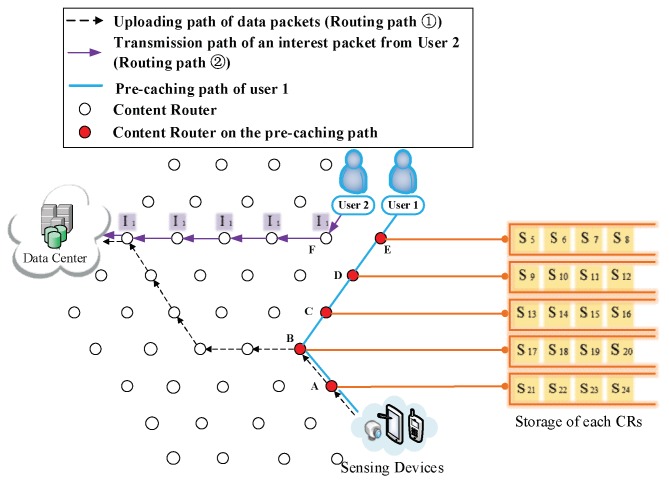
User 2 joins the network and requests the same data that User 1 had requested.

**Figure 19:**
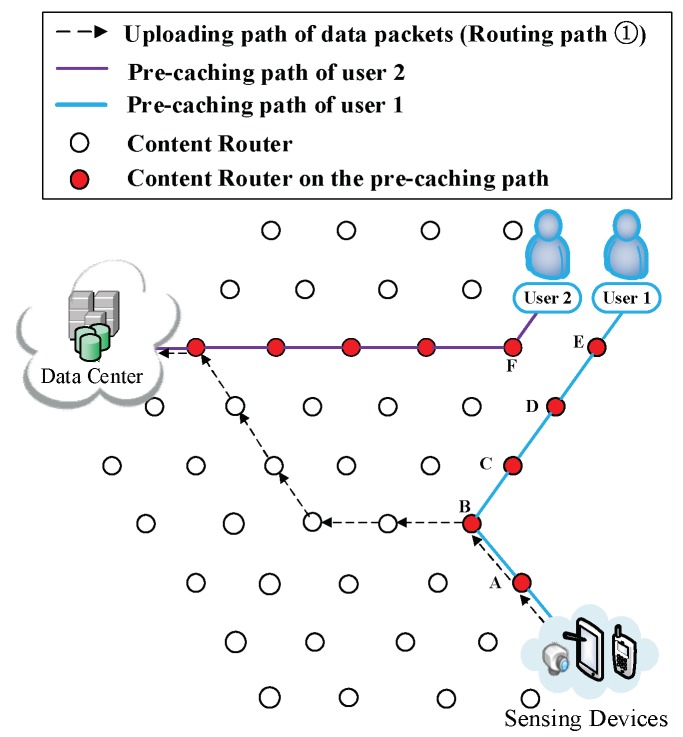
The formation of User 2′s $P_{cache}$.

Next, the whole process of the CJSR scheme is illustrated using a concrete example along with a series of detailed figures. We make some modifications to the scenario we used in Figure 1 and use it as our example. It is assumed that there are one user (another user will join the network later), one Data Producer (DP), one Data Center (DC) and n CRs. When breaking news happens, a user issues an interest for a short video about this news and hopes to obtain the data with high QoS, a DP is collecting and sensing data relevant to the news, and a DC is waiting for the data to be uploaded. Moreover, we assume that the requested streaming contains 20 data packets, $N_{\xi }$ = 5, and the storage size of each CR is 4 data packets. At the beginning, the user is interested in $S_{1}$ and requests it. The forwarding of $I_{1}$ automatically find the shortest route to a DC. To focus on routing paths, we omit the links among CRs in the following graphs. Detailed procedure and ideas are:

(1) Initial process of requesting data. In Figure 6, a DP is collecting data about that news, then uploads data to a DC according to its public prefix (Routing Path ① in Figure 6). Then, the user requests $S_{1}$. By that time, the user does not possess any information about where the data are produced, so the interest packet is sent to the DC (Routing Path ② in Figure 6). Because the requested data $S_{1}$ have not been uploaded, the DC responds with the prefix of the DP that is collecting the requested data to the user (Routing Path ③ in Figure 6). As a result, uploading pre-caching of the cooperative pre-caching mechanism is going to be performed in this example.

(2) Re-direction of data request. After receiving the prefix information about the DP, the user starts to request data from the DP directly (Routing Path ② in Figure 7).

(3) The formation of the first shortcut routing path. As it is shown in Figure 8, a transmission path between the DP and the user is built thus the user does not have to obtain data from the DC. In this case, the shortcut is also the $P_{cache}$. The number of CRs ($N_{\xi }$) on the $P_{cache}$ is five. Because of the scarcity of storage size, $S_{1}$ to $S_{20}$ is divided into five arrays (as it is demonstrated in the left part of Figure 8), then the first round of pre-caching is conducted and five arrays are cached in five CRs along the $P_{cache}$.

(4) Pre-caching on the uploading path of data. Routing Path ② in Figure 9, Figure 10, Figure 11, Figure 12 and Figure 13 demonstrates the whole procedure of uploading pre-caching. Because the subsequent part of the same streaming is likely to be request later, especially the first array. Besides $S_{1}$, $S_{2}\;\mathrm{to}\;S_{4}$ are more likely to be requested later than other arrays. Hence, the first array is assigned to Vertex E. $S_{1}$ is sent to user as soon as $S_{1}$ is received so the first request of user is satisfied by that time and the following pre-caching are carried out. $S_{5}\;\mathrm{to}\;S_{8}$ are assigned to Vertex D, of which the hop counts to user is two. Similarly, $S_{9}\;\mathrm{to}\;S_{12}$ are cached in Vertex C, of which the hop counts to user is three; $S_{13}\;\mathrm{to}\;S_{16}$ are cached in Vertex B; and $S_{17}\;\mathrm{to}\;S_{20}$ are cached in Vertex A. The storage of each CR is shown in Figure 14.

(5) The formation of the second kind of shortcut. Initially, the data are uploaded as shown in Routing Path ① of Figure 8. After the formation of the first kind of shortcut, data packets pass through the $P_{cache}$ while they are uploaded as it is depicted in Routing Path ① of Figure 9, so that they are cached into CRs while they are uploading. During the conducting of pre-caching, the back proportion of the remaining data are assigned to the CRs that are far away from user. Data packets would adjust its shortcut to find a CR that could yield shorter overall routing path, then upload data through that chosen CR to the DC. Firstly, the chosen CR is Vertex D (Figure 10). Then, the uploading path of data packets follows Routing Path ① in Figure 11. Following that, the uploading path is shown as Routing Path ① in Figure 12. Finally, data packets upload through Vertex A (Routing Path ① in Figure 13).

(6) Request of cached data (the user’s requests). Rather than forward each interest packet to DP in a time-consuming way, interest packets could be handled by CRs that have the requested data cached.

(7) The participation of other users. There may be two possible situations after User 2 joins the network: (i) User 2 requests for different data. Such a scenario is demonstrated in Figure 15, Figure 16 and Figure 17. Firstly, User 2 requests $S_{k}$ from the DC (Routing Path ② in Figure 15). It is reasonable that User 2 may request the other relevant data of that breaking news (also have not been uploaded yet) from another DP. Since the requested data are different, two users would conduct the CJSR scheme separately. Because User 1 and User 2 are both located on the network edge, it is assumed that they are connected to Vertex E and Vertex F respectively. After requesting from the DC, User 2 gains the prefix information of DP 2 and sends request to it (a new caching path is established as shown in Caching Path ② in Figure 16). After constructing this pre-caching path, the two users’ pre-caching path overlap at Vertex C as demonstrated in Figure 17. When User 2 starts to request data, a new round of pre-caching is triggered, and all the data cached in the overlapped CR (Vertex C) would be replaced. This scenario is evaluated in Scenario 4 in Section 5. (ii) User 2 requests for data that has been uploaded. Figure 18 and Figure 19 depict that User 2′s request are satisfied by the DC, although the requested data may still be held by a CR. Since the first interest packet of User 2 is forwarded to the DC, the pre-caching path is built between the DC and the User 2 and the downloading pre-caching is triggered. Following that, the DC would perform downloading pre-caching (similar procedures demonstrated in Figure 9, Figure 10, Figure 11, Figure 12 and Figure 13 are omitted).

### 4.3. Idle CRs Involved Pre-Caching (ICIP)

In Algorithm 1, data packets are assigned to CRs along the $P_{cache}$, which could be downloading routing paths from DCs to users or uploading routing paths from DPs to users. However, during the whole process of the CJSR scheme, only those CRs on those fixed paths are actively involved in pre-caching. To exploit the storage resource from underused idle CRs (we define a CR that is not on the $P_{cache}$ as an idle CR) and help CRs on the caching path to cache the data that they cannot accommodate, Algorithm 3 is proposed. In this algorithm, Step 1 is about processing an interest packet. What is different from Algorithm 1 is that the frequency of an interest packet is recorded and used as the criteria in judging the importance of its corresponding data. Step 2 describes the procedures of processing a data packet at each CR.

The main idea in Algorithm 3 is to address the scarce caching storage in an indirect method. In the first place, a standard should be made to judge the priority of a data packet. If the number of interest packets requesting for a data packet is high, these data are more popular, and may be related to a hot issue or breaking news. Therefore, each CR records a frequency of an interest packet ($A_{\xi_{i,}r_{j}}^{m}$) and uses it as the criterion. For a newly arrived data packet, comparisons of the Interest frequency are being made before asking help from an idle CR. If the Interest frequency of the incoming data are higher than that of one or more stored data packets, the least frequently used data packet is replaced by the new one. If all the Interest frequency of cached data packets are higher than that of the incoming data packet, which means that all of the stored data have high probability to be requested sooner, this CR will ask an adjacent idle CR to store that unaccommodated data. Here is the detailed process: after a period of pre-caching, the storage of each CR on the $P_{cache}$ is full. When a new data packet arrives at $\xi_{ij}$, the $A_{\xi_{i,}r_{j}}^{m}$ of each cached data packet is compared and the least frequent used data are replaced. If the $A_{\xi_{i,}r_{j}}^{m}$ of cached data packets are all high, the newly arrived data packet is forwarded to an adjacent idle CR $\xi_{idle}$, and store ename and extra chunks into the ES. When user requests that data, CRs would check the ES of their adjacent CRs if the data are not found after performing traditional lookup. Using the PathMark field of data packets, if a CR cannot accommodate a data packet, the original sequence number indicating its destination is modified into a special mark, then this data packet is broadcasted to neighbors. Because no CR along the caching path matches the PathMark field of the data packet, this data packet is immediately dropped and only an idle CR could cache it. The detailed process is shown in the following Algorithm 3.


**Algorithm 3 Idle CRs Involved Provident Caching (ICIP)**
1:$I_{m}$ requesting for $S_{m}$ from $S_{n}$*=* {$S_{1},S_{2},\ldots ,S_{m},\ldots ,\;S_{n}$} is synthesized by user $r_{j}$
2:**Step 1**: $I_{m}$*arrives at*$\xi_{ij}$3:$A_{\xi_{ij,}r_{j}}^{m}$++4:**If**$S_{m}$ is found after performing lookup in the Content Store5: Forward $S_{m}$ to $r_{j}$
6:
**Else**
7: Check ES8: **If** found entry in ES9:  Forward $S_{m}$ to $r_{j}$
10: **Else** forward $I_{m}$ to next vertex11:  **If**
$I_{m}$ arrives at a DC or a DP12:   Perform algorithm 113:  **End If**14:  **End If**15:
**End If**
16:
**Step 2:**
*A data packet*
$S_{k}$
*arrives at a CR*
$\xi_{ij}$
17:**If**$I_{k}$ is found in the PIT of $\xi_{ij}$
18: **If** the $P_{m}$ of $\;S_{k}$ is identical to $\xi_{ij}$’s sequence number19:   **If** the left cache storage of $\xi_{ij}$ is enough20:  Cache the $S_{k}$
21:  **Else**22:  **For** each cached data packet $S_{i}$ in $\xi_{ij}$
23:    **If**
$A_{\xi_{ij,}r_{j}}^{k}>A_{\xi_{ij,}r_{j}}^{i}$
24:   Replace the least frequently used data packet $S_{i}$ with $S_{k}$
25:   **Else**26:   Forward the $S_{k}$ to $\xi_{idle}$
27:   Add $S_{k}$ to Extra Store 28:   **End If**29:  **End For**30:   **End If**31: **End If**32:
**Else**
33: Drop $\;S_{k}$
34:
**End If**


The procedure of Algorithm 3 is also illustrated using an example, in which the assumptions and parameters are same as they are in Section 4.1.

**Figure 20:**
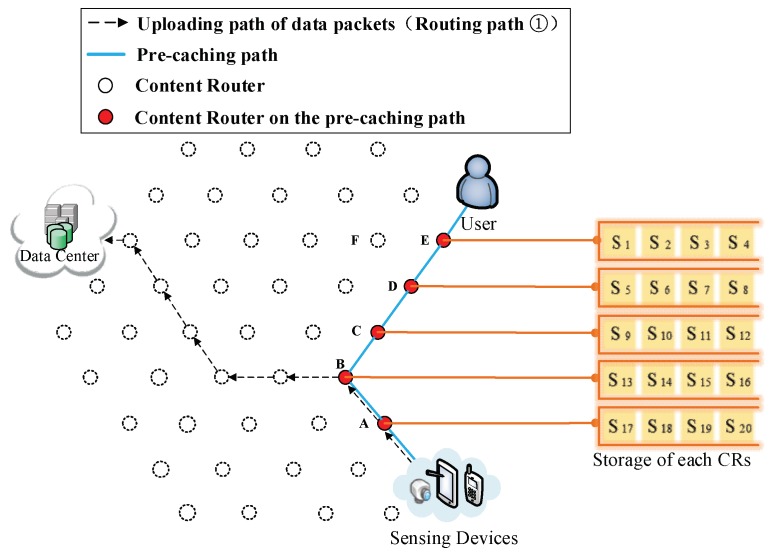
Cache capacity of each CR is full.

**Figure 21:**
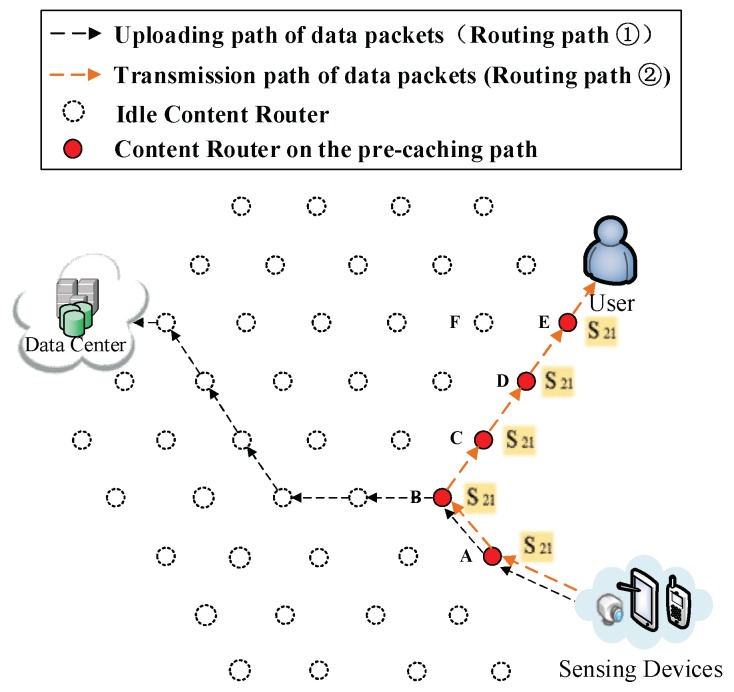
$S_{21}$ is forwarded to Vertex E.

**Figure 22:**
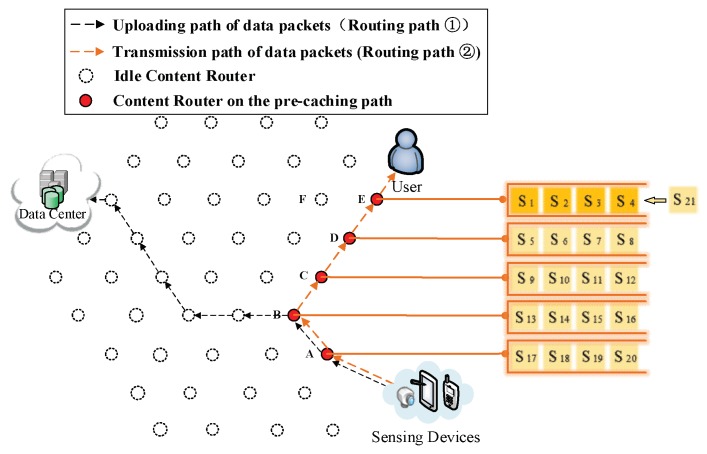
Comparisons of the Interest frequency.

**Figure 23:**
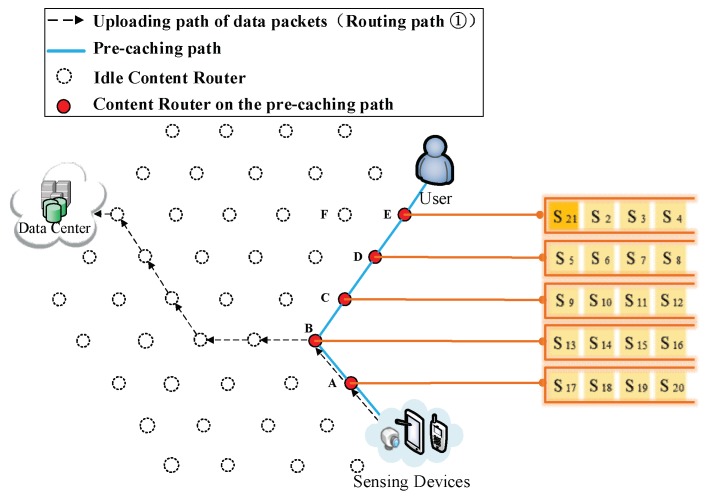
Replacement of $S_{1}$.

**Figure 24:**
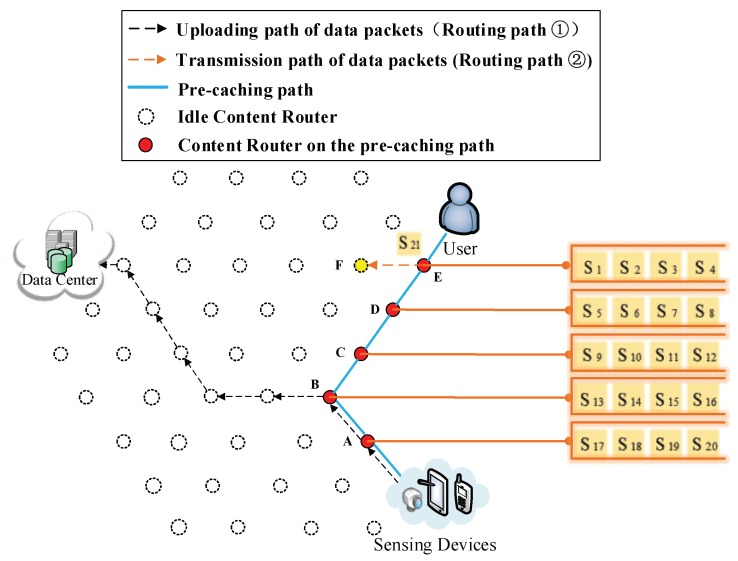
Forward $S_{21}$ to Vertex F.

**Figure 25:**
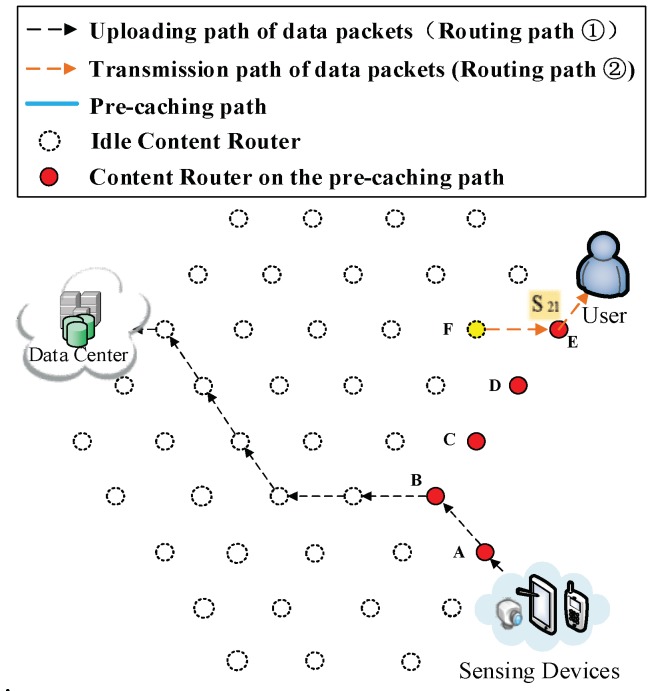
Forward $S_{21}$ in respond to the user.

(1) Pre-caching process. After performing uploading pre-caching for a period, due to the limitation of caching storage (four data packets), storage capacity of each CR has been filled, as Figure 20 demonstrates.

(2) Replacement of a data packet. At some time while the DP is conducting pre-caching, the data packet $S_{21}$ is assigned to Vertex E (Routing Path ② in Figure 21). Figure 22 demonstrates that, when $S_{21}$ arrives at Vertex E, the $A_{E,r_{j}}^{1},\;A_{E,r_{j}}^{2},\;A_{E,r_{j}}^{3}\;\mathrm{and}\;A_{E,r_{j}}^{4}$ are compared with $A_{E,r_{j}}^{21}$, respectively. As illustrated in Figure 23, the result of comparisons is that $A_{E,r_{j}}^{1}$ < $A_{E,r_{j}}^{21}$, then $S_{1}$ is substituted by $S_{21}$.

(3) Using storage capacity of an adjacent idle CR. If all the Interest frequency of cached data packets in Vertex E ($A_{E,r_{j}}^{1},\;A_{E,r_{j}}^{2},\;A_{E,r_{j}}^{3}\;\mathrm{and}\;A_{E,r_{j}}^{4}$) are bigger than $A_{E,r_{j}}^{21}$, $S_{21}$ is forwarded to Vertex E (Routing Path ② in Figure 24). When $S_{21}$ is requested, the Vertex F sends $S_{21}$ to the user according to the Extra Storage (Routing Path ② in Figure 25).

## 5. The Experimental Results and Analysis

In this section, extensive experiments were conducted and the performance of the CJSR scheme is evaluated under three different metrics. We made comparisons among two proposed schemes and five existing schemes: (1) Traditional NDN; (2) No Cache; (3) Probability Cache; (4) LCD; and (5) MCD. In the traditional NDN scheme, data packets are cached into each CR they have been forwarded. In contrast, CRs in the No Cache scheme can only forward packets. As for the probability Cache scheme, a CR will cache a data packet based on a predefined probability; the probability parameter is set to 0.5 in our experiments. The LCD and the MCD scheme share a similar idea which is to cache the data packet in the next vertex of the vertex where a cache hit occurs. However, in the latter scheme, the hit data cached in the CR where the cache hit takes place is deleted. Initially, the total hop counts are compared and analyzed in Section 5.1, which denotes the number of total hop counts of all the requested data packets. This criterion reflects the latency and is of critical importance to meet real-time requirement of many applications. Next, the number of processed packets on each CR along the caching path is evaluated in Section 5.2. The processed interest packets and the processed data packets denote the number of forwarded interest packets and incoming data packets respectively. Finally, comparisons of cache hits are made in Section 5.3.

After analyzing the experiment results, we have concluded that for the optimization targets Equations (1)–(3), the proposed mechanism guaranteed high performance in terms of the amount of processed packets, cache hits and total hop counts. High performance of these metrics above would contribute to a high QoS for users.

**Figure 26:**
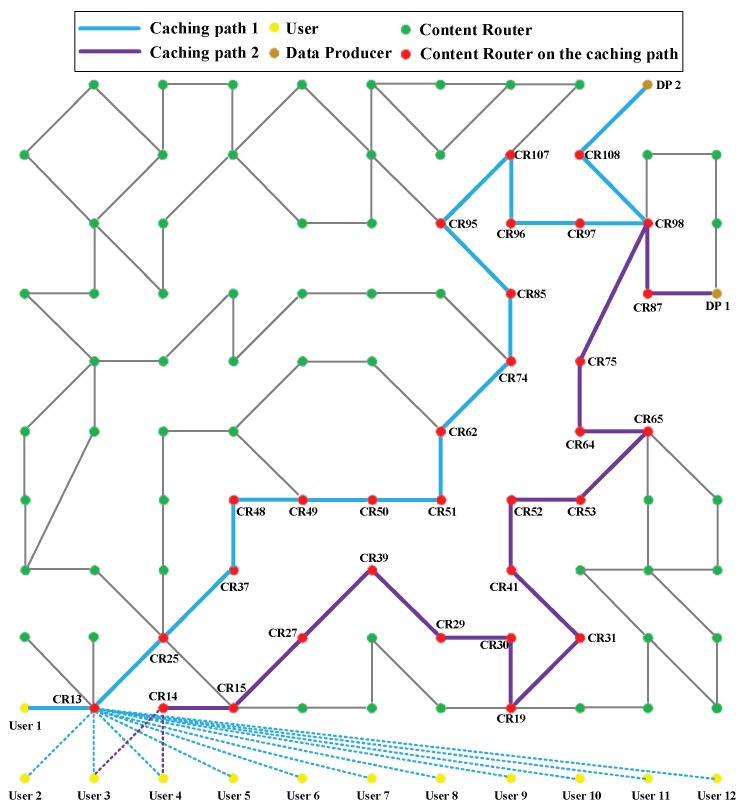
Network topology for Scenarios 1–5.

**Figure 27:**
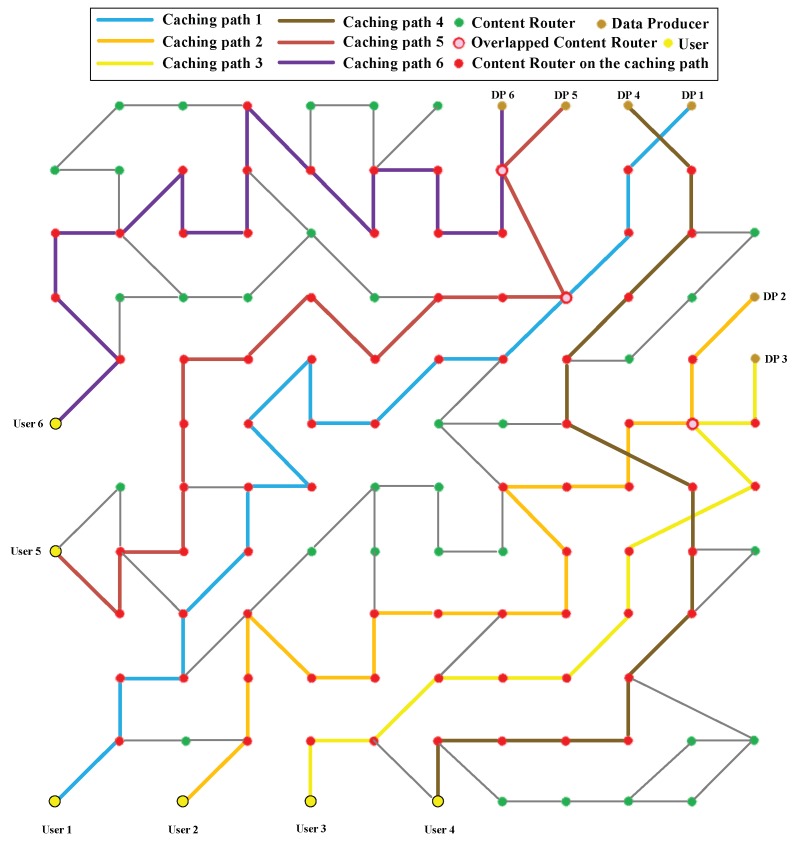
Network topology for Scenario 6.

To test the proposed CJSR scheme under various scenarios, a topology deployed with 88 Content Routers (Figure 26) is produced for the experiments. The dotted lines denote that Users 2–12 are connected to different CRs in various scenarios. As shown in Figure 27, another larger topology (113 Content Routers) is generated for Scenario 6. Overall, to evaluate the uploading pre-caching of the CJSR scheme, all of the scenarios assume the following: (i) When users request data, their interested data have not been uploaded yet, so the first kind of shortcut is built between the DP and users; to focus on the routing between DPs and users, DCs are omitted in the topology. (ii) All the CRs are available and willing to cooperate. (iii) All users request successively and continuously.

Next, we discuss the design and configurations of the experiments. To test the network performance under various situations, six scenarios are designed. The detailed parameters and configurations can be found in Table 2 and Table 3. Overall, the cache capacity is set to 250 so that each CR can accommodate 250 data packets, and the LRU replacement policy is used to replace old content in cache storage. In addition, each chunk has a Time-To-Live (TTL) to limit its lifespan if necessary. However, the configuration of TTL is highly application-dependent and the detailed evaluation on it is beyond the scope of this work. Hence, the TTL used in the experiments is set to be larger than 5 s (which is the time from a chunk is cached to it is replaced, so that each chunk remains stored in cache until it is replaced). The requested data contain 30,000 data packets, the interest frequency is 50 interests per second and simulation time is 10 min.

Scenario 1: In this scenario, one user is deployed. Experiment results reveal that most of interest packets are processed by intermediary CRs. However, the amount of processed data only had a slight decline because the assignments of data would produce some traffic. However, when more than one user request the same data, as in Scenario 3, the traffic of data packets would decline because data assignments only have to occur once, then only the transmission of data packets from CR holding the requested packets to users would produce some traffic.

Scenario 2: User 1 and User 2 request same data from DP 1. User 2 starts to request one second later than User 1. Requesting later, User 2 can directly benefit from the cached data. In the beginning of the second round pre-caching, while User 2 requests $S_{3949}$ to $S_{3999}$, those data packets have just been replaced by the assigned data from User 1 ($S_{4000}$ to $S_{4049}$) prior to User 2’s request. Therefore, User 2 has to send $I_{3949}$ to $I_{3999}$ to the DP. Similarly, User 2′s requests have to be forwarded to the DP in the remaining rounds (the whole process consists of eight rounds of pre-caching). In contrast, however, the request of replaced data from User 2 ($S_{3949}$ to $S_{3999}$, $S_{7949}$ to $S_{7999}$ etc.) from User 2 can be satisfied in the ICIP scheme due to the involvement of idle CRs. Because each user requests consecutive data, the interest frequency of data packets are same. As a result, when the assigned data from User 1 arrives at CR13, no replacement occurs and CR13 forwards newly arriving data packets (e.g., $S_{4000}$ to $S_{4049}$) to an idle CR. When User 2 requests $S_{3949}$ to $S_{3999}$, his interest can still be satisfied. Nevertheless, if User 2 starts to request five seconds later, which is the time that cache replacements occur, User 2 cannot benefit from cached data at all.

Scenario 3: Twelve users (Users 1–12) request same data from DP1. After the first user discovers data relevant to breaking news, same data may be requested by many users at the same time. This scenario depicts such a situation and Users 2–12 are started at the same time one second latter than User 1. In this scenario Caching Path 2 is choose to evaluate performance.

Scenario 4: Two users request different data from two DPs. In this scenario, users conduct their own caching schemes separately. Nevertheless, when the CJSR and the ICIP schemes are applied, the caching paths of two users may overlap because the form of caching path is the travelled path of the interest that triggers the pre-caching. As shown in Figure 26, the two caching paths overlapped at CR 98; as a result, the overlapped CR has to undergo more traffic burden. The overlap would also have negative influence for the CJSR scheme. In each round of pre-caching, the data packets assigned to the CR 98 are replaced by each other, so that interest packets cannot be satisfied by CR 98; then they are forwarded to the DP; and LCE cache policy is used for the remaining data which could have been satisfied by the CR 98 if there were only one user. Following that, all the cached data of CRs along the caching path was replaced due to those requests. For instance, in the first round, $S_{0}$ to $S_{3499}$ from User 1 and User 2 are satisfied by assigned data. Next, $I_{3499}$ to $I_{3749}$ have to be sent to the DP, and all the cached data packets of CRs along the caching path are replaced by $S_{3499}$ to $S_{3749}$ because they are sent back using LCE policy. Then, the remaining requests of first round ($I_{3749}$ to $I_{3999}$) have to be sent to the DP. Therefore the overlapped CR has a negative impact on the CJSR scheme, however, the ICIP scheme is not influenced. Conversely, during the data assignments of the ICIP scheme, the conflicts of data replacement are solved by the involvement of an idle CR. Thus, both users enjoy assigned data throughout each entire round. Caching Path 2 is used to demonstrate results.

Scenario 5: User 1 and User 2 request same data from DP 1 while User 3 and User 4 are requesting same data from DP 2. User 1 as well as User 3 start to request at the same time, then User 2 and User 4 are started one second later. Similar to Scenario 4, the rest of interest packets are forwarded to the DP from the moment their interest packet arrives at CR 98.

Scenario 6: To comprehensively test the proposed algorithms, another topology is used in this scenario as shown in Figure 27. In addition, there are six DPs producing various data for six users. While the CJSR scheme is conducted, six caching paths are built separately. Similar to Scenario 4, sometimes caching paths overlap and the CJSR scheme would not perform as good as it does in other scenarios due to the conflicts of data replacement. However, those conflicts have no impact on the ICIP scheme. Finally, Caching Paths 3, 4 and 6 are chosen to depict the experimental result because they represent different length and two of them contain CRs that shared by multiple caching paths.

### 5.1. Delay Analysis

**Figure 28:**
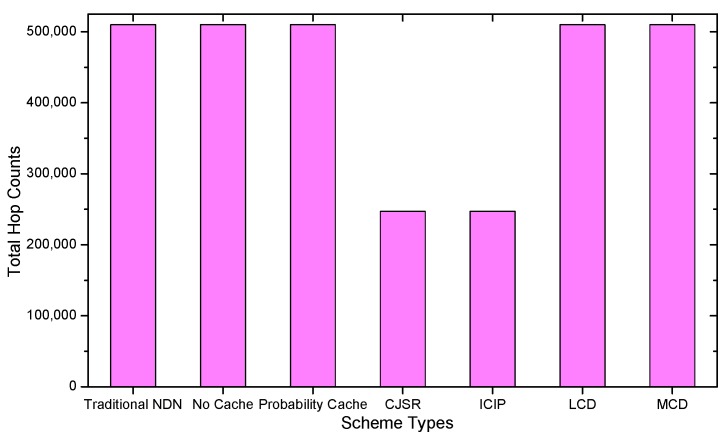
The total hop counts of all the requested data packets in Scenario 1.

**Figure 29:**
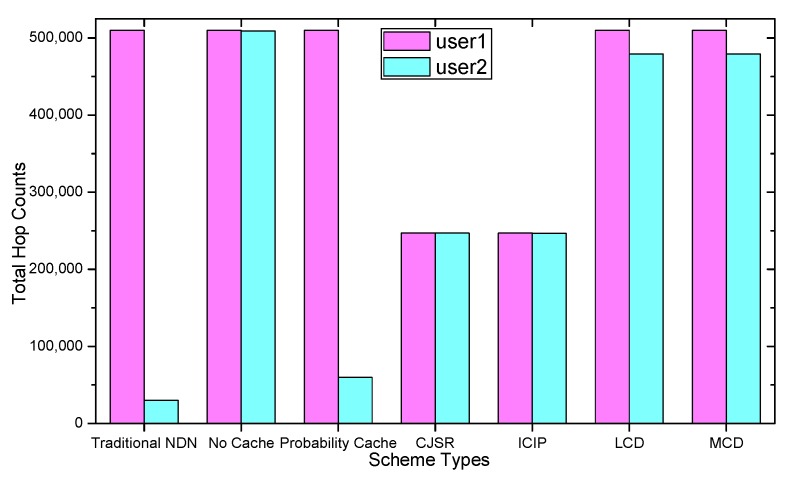
The total hop counts of all the requested data packets in Scenario 2.

**Figure 30:**
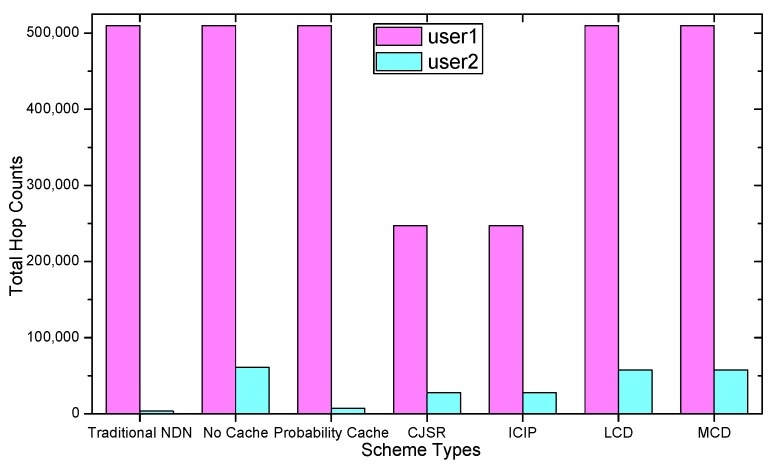
The total hop counts of all the requested data packets in Scenario 3.

**Figure 31:**
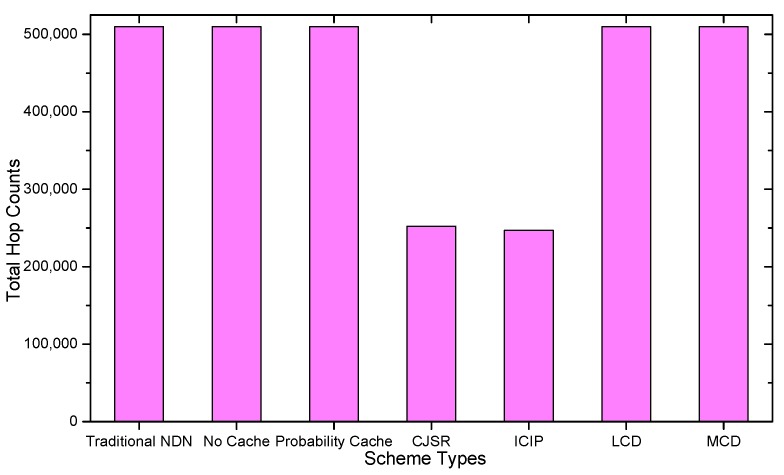
The total hop counts of all the requested data packets in Scenario 4 for both User 1 and User 2.

**Figure 32:**
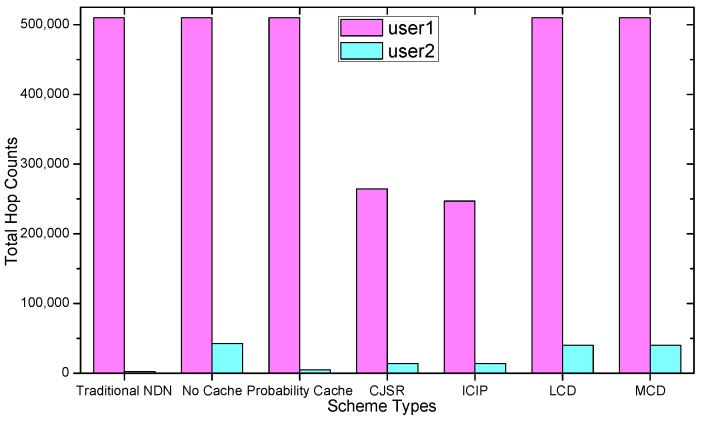
The total hop counts of all the requested data packets in Scenario 5.

**Figure 33:**
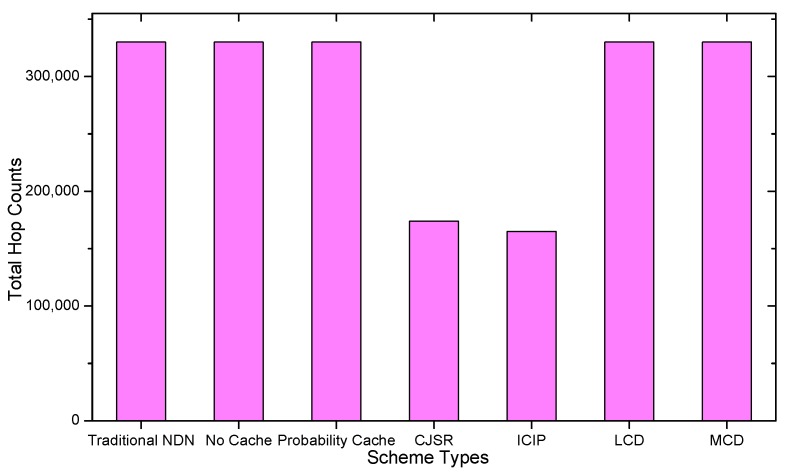
The total hop counts of all the requested data packets in Scenario 6 for User 3.

**Figure 34:**
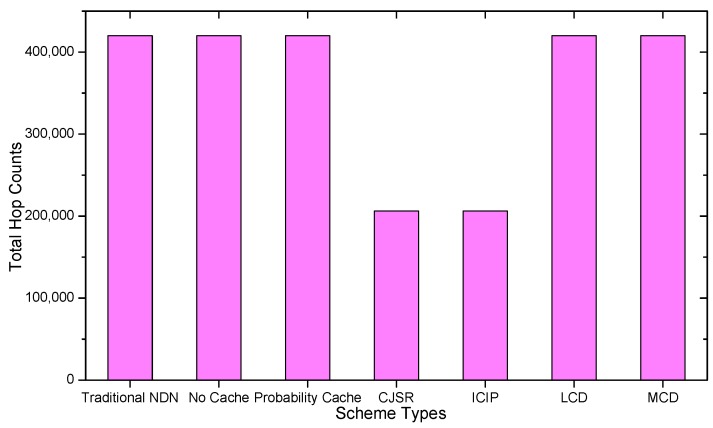
The total hop counts of all the requested data packets in Scenario 6 for User 4.

**Figure 35:**
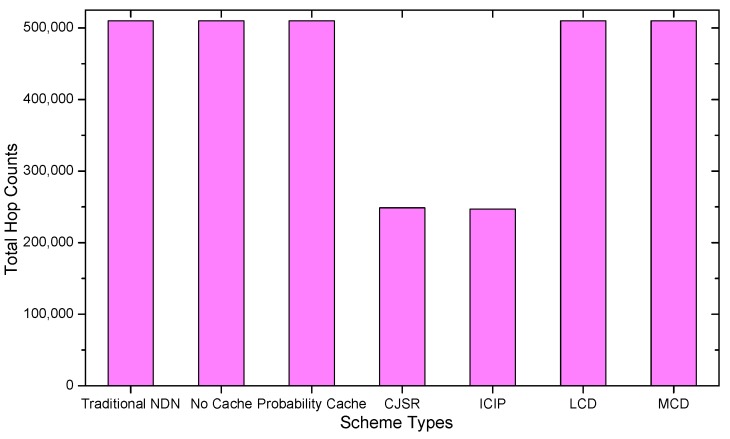
The total hop counts of all the requested data packets in Scenario 6 for User 6.

As shown in the Figure 28, Figure 29, Figure 30, Figure 31, Figure 32, Figure 33, Figure 34 and Figure 35, the given bar charts illustrate that the CJSR scheme reacts to user’s request swiftly and the total hop counts of User 1 has reduced 51.6% compared with that of other schemes. According to Figure 29, Figure 30 and Figure 32, when there are more than one users, User 2′s total counts of the traditional NDN outperformed proposed schemes because User 2′s requests are satisfied by $\xi_{r_{1}}$(CR13) which has one hop count to the users. With lower total hop counts, high QoS could be guaranteed and the optimization objective of Equation (2) is achieved.

### 5.2. Packets Processed Analysis

**Figure 36:**
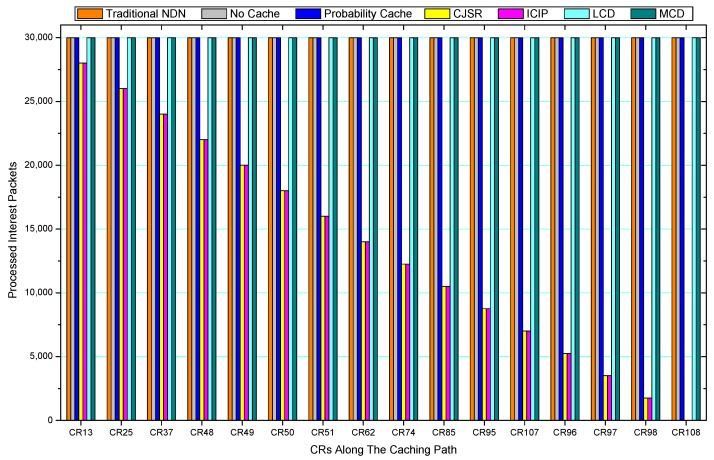
The number of processed interest packets of each CR along Caching Path in Scenario 1.

**Figure 37:**
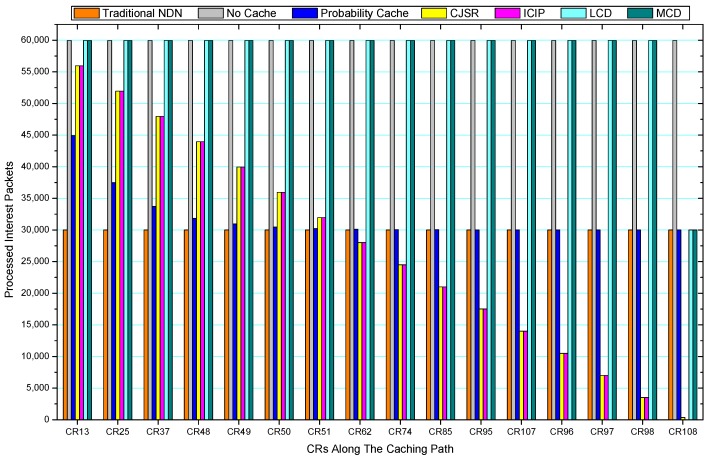
The number of processed interest packets of each CR along Caching Path in Scenario 2.

**Figure 38:**
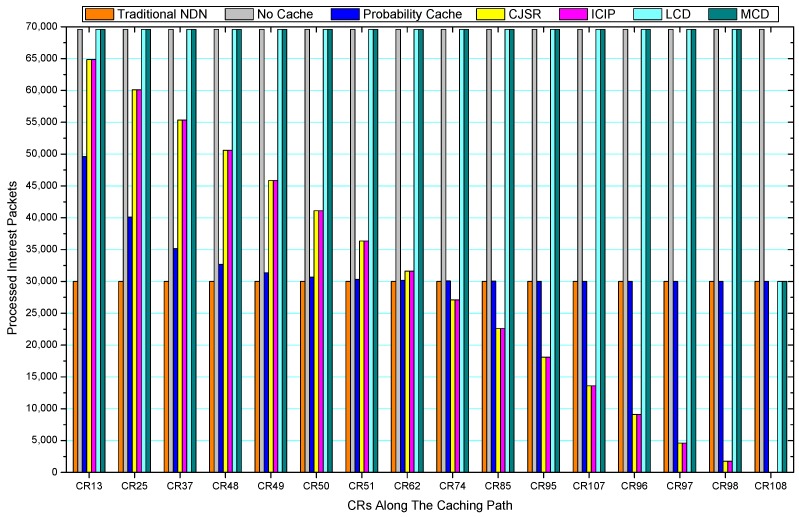
The number of processed interest packets of each CR along Caching Path in Scenario 3.

**Figure 39:**
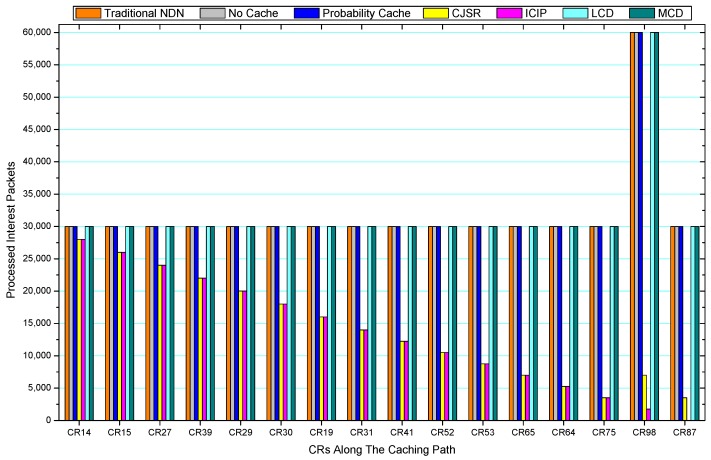
The number of processed interest packets of each CR along Caching Path 1 in Scenario 4.

**Figure 40:**
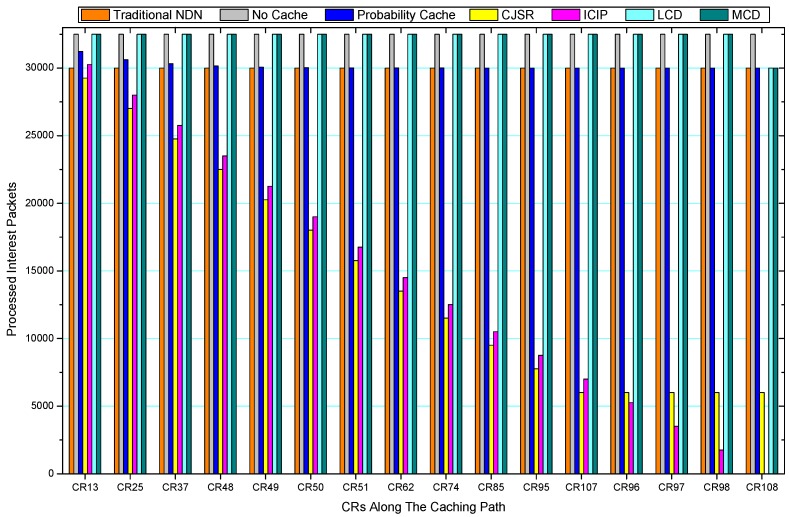
The number of processed interest packets of each CR along Caching Path 1 in Scenario 5.

**Figure 41:**
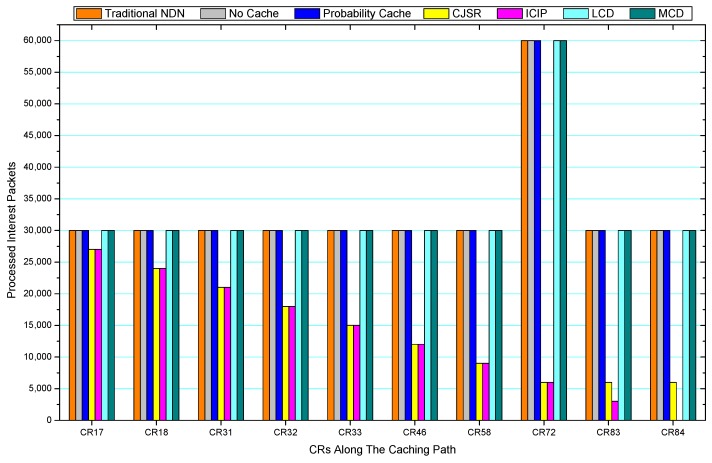
The number of processed interest packets of each CR along Caching Path 3 in Scenario 6.

**Figure 42:**
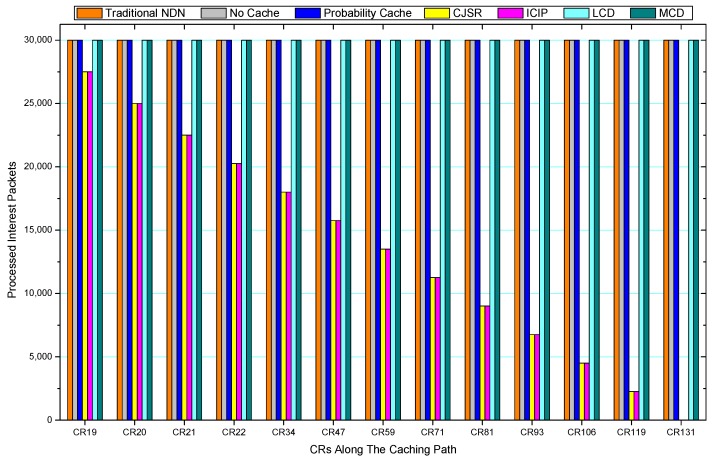
The number of processed interest packets of each CR along Caching Path 4 in Scenario 6.

**Figure 43:**
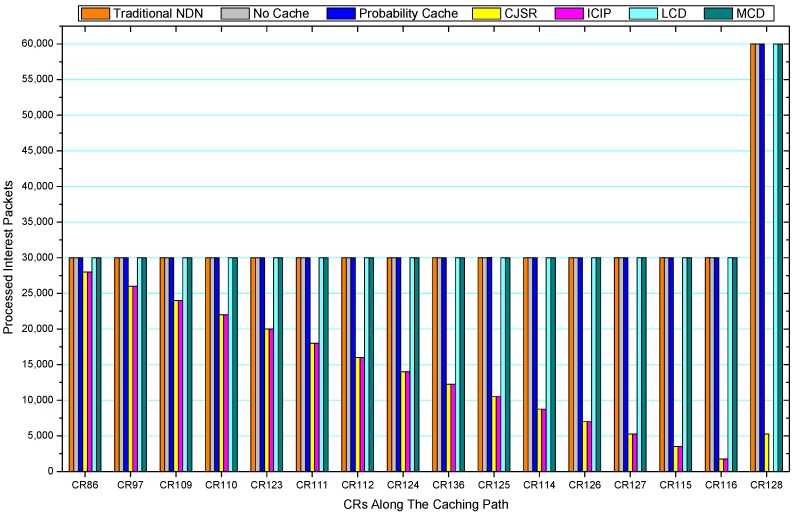
The number of processed interest packets of each CR along Caching Path 6 in Scenario 6.

**Figure 44:**
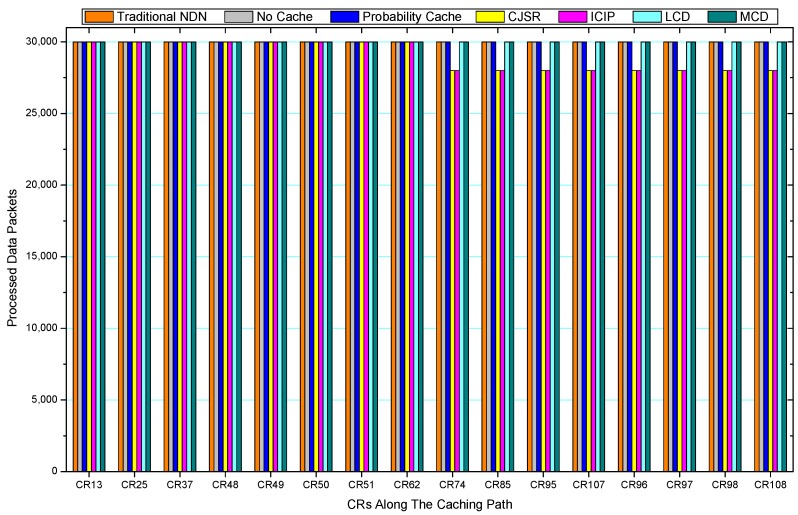
The number of processed data packets of each CR along Caching Path in Scenario 1.

**Figure 45:**
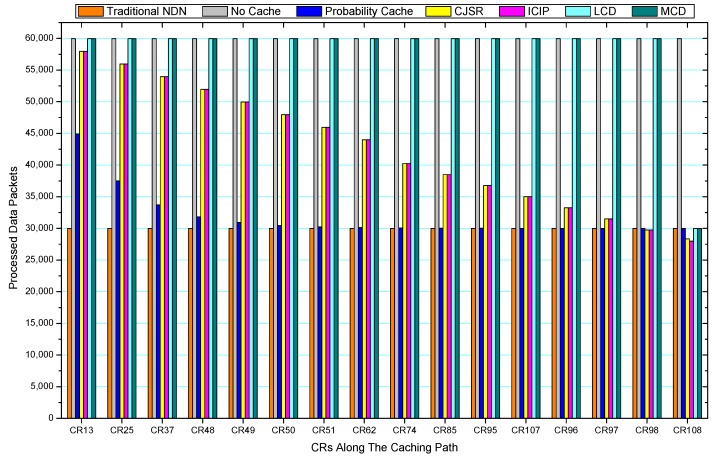
The number of processed data packets of each CR along Caching Path in Scenario 2.

**Figure 46:**
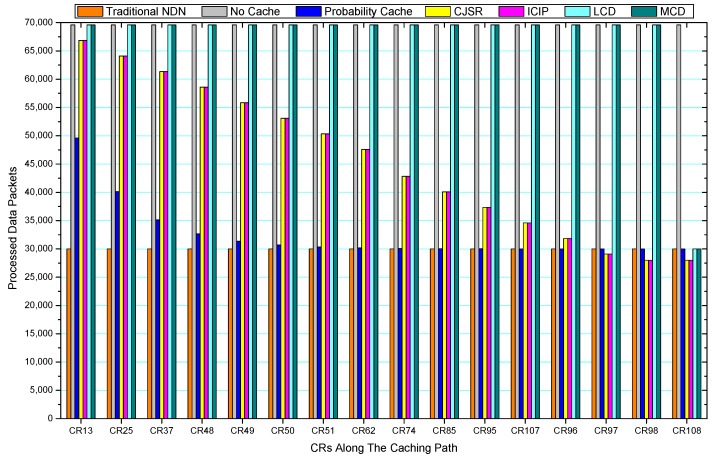
The number of processed data packets of each CR along Caching Path in Scenario 3.

**Figure 47:**
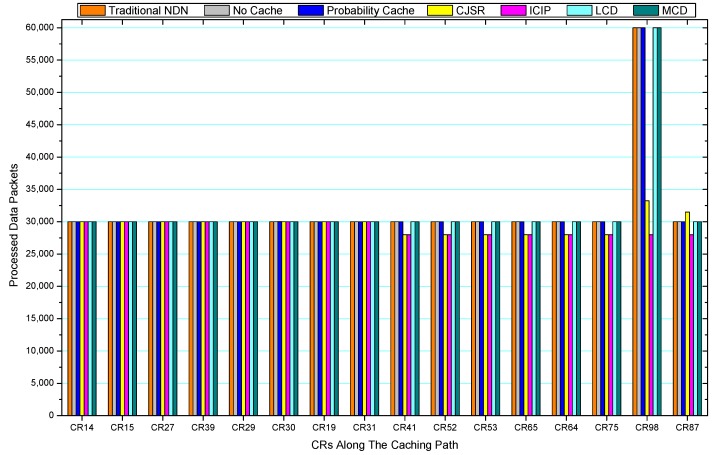
The number of processed data packets of each CR along Caching Path 2 in Scenario 4.

**Figure 48:**
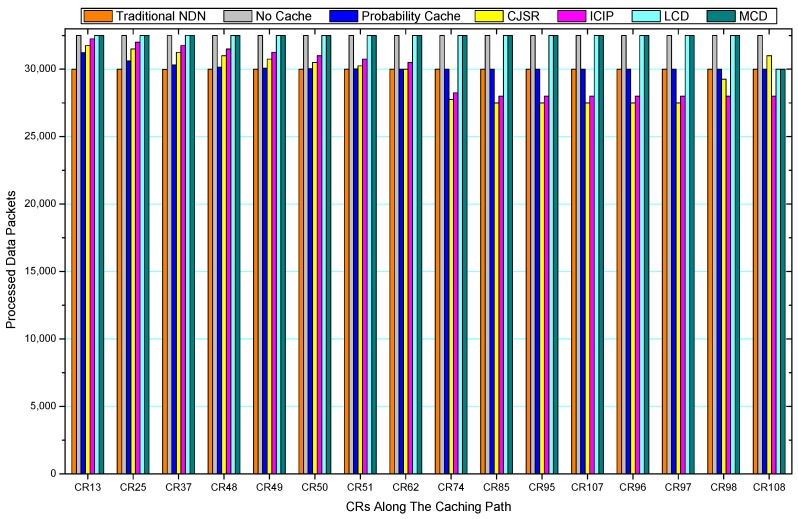
The number of processed data packets of each CR along Caching Path 1 in Scenario 5.

**Figure 49:**
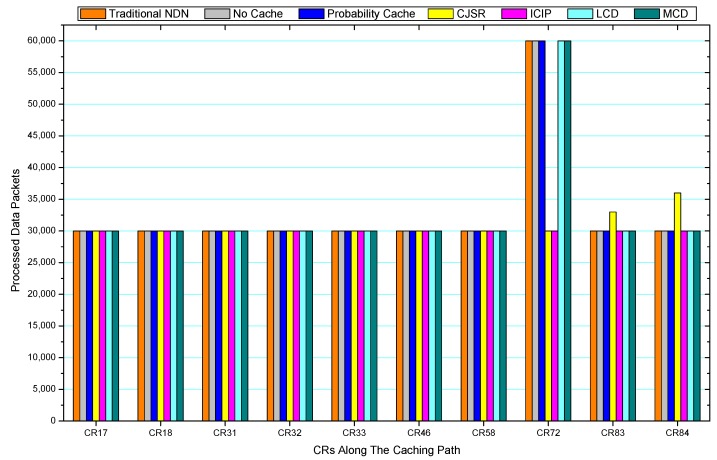
The number of processed data packets of each CR along Caching Path 3 in Scenario 6.

**Figure 50:**
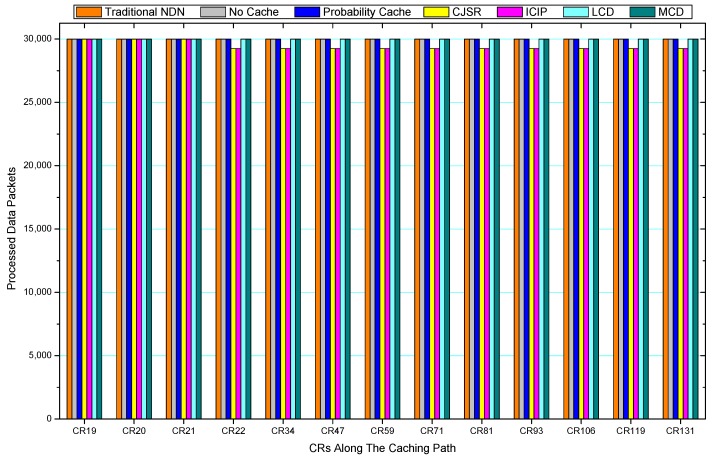
The number of processed data packets of each CR along Caching Path 4 in Scenario 6.

**Figure 51:**
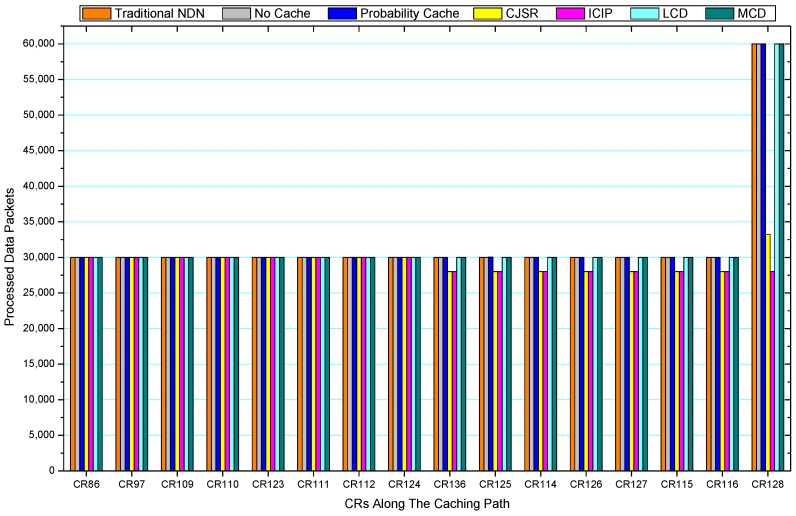
The number of processed data packets of each CR along Caching Path 6 in Scenario 6.

Figure 36, Figure 37, Figure 38, Figure 39, Figure 40, Figure 41, Figure 42, Figure 43, Figure 44, Figure 45, Figure 46, Figure 47, Figure 48, Figure 49, Figure 50 and Figure 51 demonstrate the overall traffic of each scheme. The comparisons are made among the number of processed interest packets and data packets of CRs along the caching path. Generally, the traffic burden of the network saw a significant decline after conducting the CJSR scheme. The existing schemes have similar high amount of processed packets, every produced packet has to be processed if there is only one user, and this would inevitably result in high burden on each vertex. Conversely, the processed packets in the proposed schemes are considerably lower, only a small proportion of total produced packets have to be processed. As shown in Figure 36 and Figure 44, the traffic of interest packets had a noticeable drop after conducting pre-caching. The ladder-shaped lines in Figure 36 clearly demonstrate that the vast majority of interest packets are processed by intermediary CRs. The chief reason is that in our schemes, the interest packets only need to flow to the CRs which have cached the requested data. As a result, the introduction of the CJSR scheme could reduce the number of processed interest and data packets on each vertex. Thereafter, the release of traffic burden leads to better User experience. It is also noticeable that the traditional NDN scheme and the Probability Cache scheme nearly perform as bad as No Cache scheme.

In conclusion, vertices that are closer to the user usually had been accessed more frequently in the CJSR scheme and the ICIP scheme, while the burden for the other three schemes is high and remain unchanged as the hop counts get larger. Seen together, the line charts reveal that, in our schemes, the burden of DP or DC was partially taken by CRs. As a result, the burden of dealing with endless packets are reduced and the users get better QoS. The reason is that, in the proposed schemes, interest packets only have to be forwarded to CRs with the corresponding data packets cached. On the contrary, the remaining schemes always have higher number of access because that interest packets have to be forwarded by each CR because data packets are not providently cached for user to fetch.

### 5.3. Cache Hits Analysis

**Figure 52:**
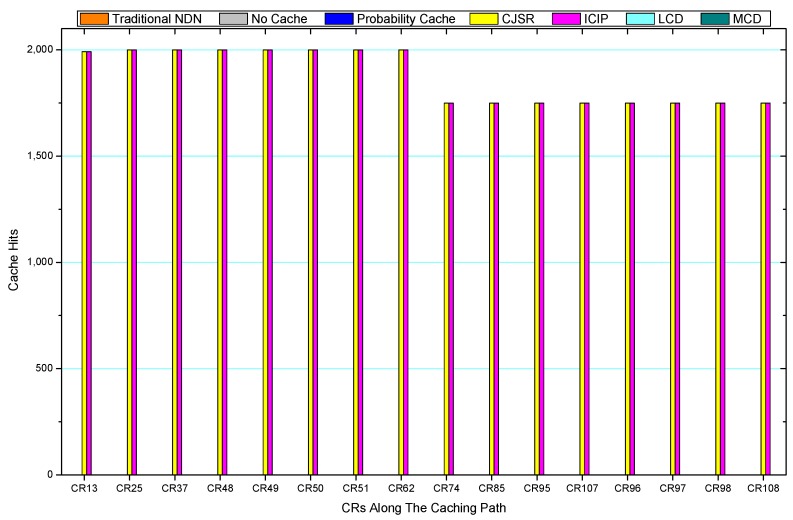
The number of cache hits on each CR along the caching path in Scenario 1.

**Figure 53:**
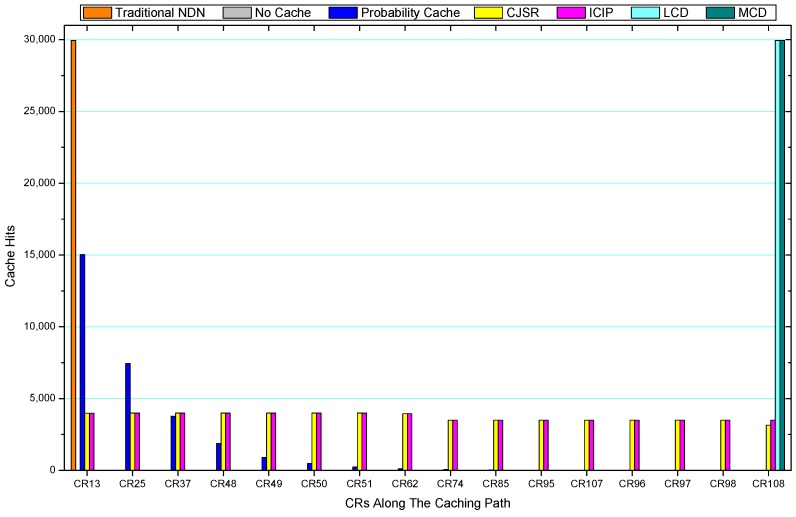
The number of cache hits on each CR along the caching path in Scenario 2.

**Figure 54:**
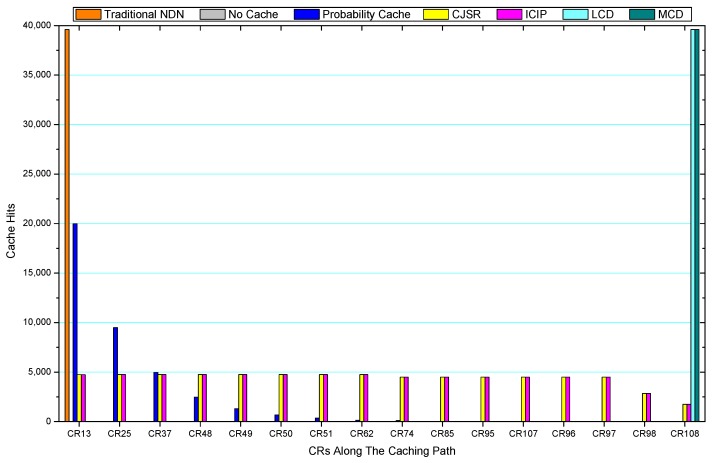
The number of cache hits on each CR along the routing path in Scenario 3.

**Figure 55:**
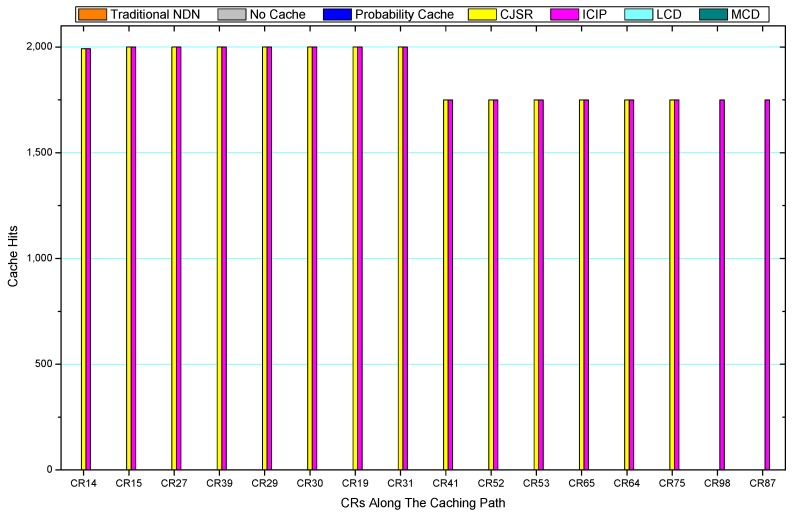
The number of cache hits on each CR along Caching Path 1 in Scenario 4.

**Figure 56:**
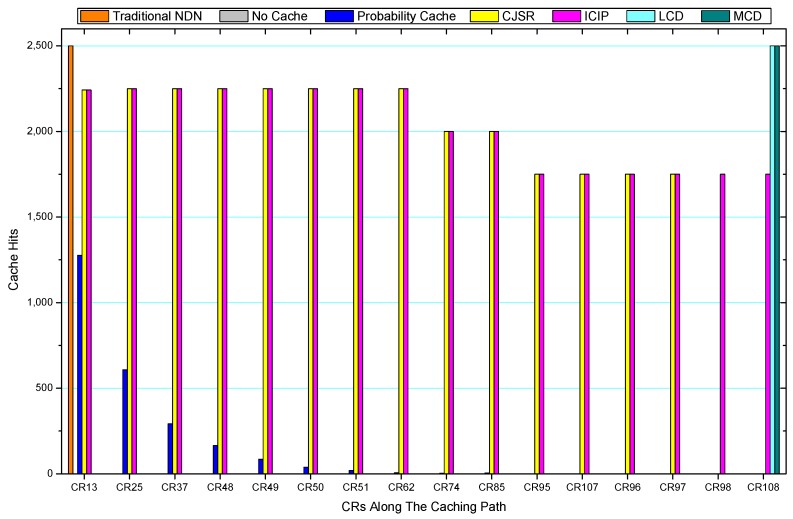
The number of cache hits on each CR along Caching Path 1 in Scenario 5.

**Figure 57:**
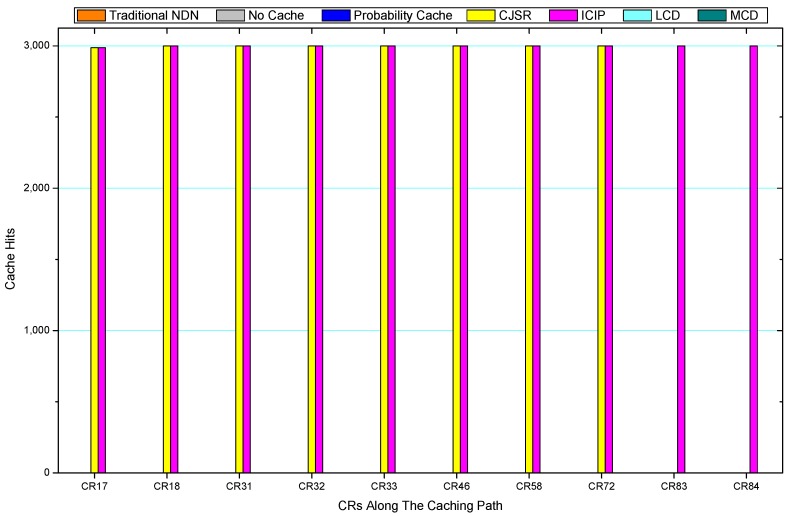
The number of cache hits on each CR along Caching Path 3 in Scenario 6.

**Figure 58:**
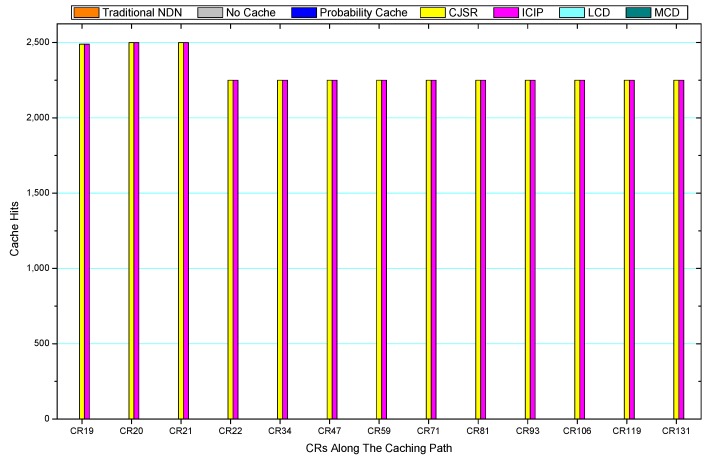
The number of cache hits on each CR along Caching Path 4 in Scenario 6.

**Figure 59:**
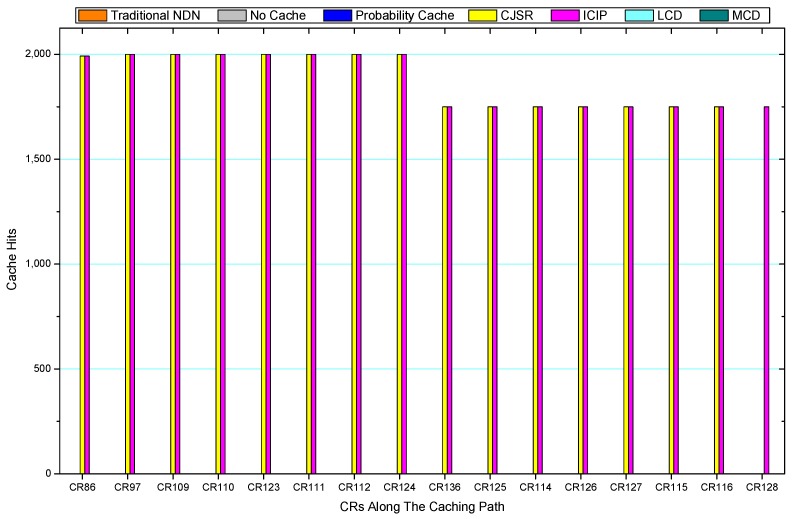
The number of cache hits on each CR along Caching Path 6 in Scenario 6.

Comparisons between our schemes and the existing caching strategies are depicted in Figure 52, Figure 53, Figure 54, Figure 55, Figure 56, Figure 57, Figure 58 and Figure 59. Figure 52 demonstrates that proposed schemes’ cache hits noticeably outnumber the remaining policies. As illustrated in Figure 53, Figure 54 and Figure 56, all the cache hits of the traditional NDN scheme occurred at $\xi_{r_{1}}$ (CR 13) and all of cache hits of the LCD and MCD schemes took place at $\xi_{p_{i}}$ (CR 108). Figure 57, Figure 58 and Figure 59 also show that all the CRs along the $P_{cache}$ have high cache hits because latter requests from the same user could benefit from providently cached data. As for the other schemes which have no cache hits at all, each CR only forwards packets. Because data packets are cached to CRs near the users intelligently. Due the involvement of idle CR, the conflicts of data replacement are solved. Thus, cache hits occurred throughout each entire caching path in the ICIP scheme. On the contrary, the DP in the remaining schemes only start to generate data packets when an interest packet is received, then the DP forwards the requested data to users in a time-consuming way.

## 6. Conclusions and Future Works

With rapid advances in the manufacturing of sensing devices, the number of data produced by ubiquitous sensing devices grows exponentially and causes heavy burden on the whole network. Aiming to minimize the hop counts for users to obtain data, reduce delay and release traffic burden, a CJSR scheme is proposed in this paper. The overall length of routing paths is reduced by using two shortcut routing methods. Furthermore, a cooperative pre-caching is proposed to decrease delay and traffic burden. One such scheme is also applicable to different situations. If the requested data have not been uploaded, users would directly request from DPs and trigger uploading pre-caching. If the data have already been uploaded, DCs would perform downloading pre-caching. The cooperative pre-caching would cache data providently into nearby CRs, thus users can directly fetch their interested data from CRs. Since the data can be found in intermediary CRs, the interest packets requesting for data are processed by CRs with the corresponding data cached. Therefore, compared with the traditional NDN scheme, the CJSR scheme could reduce the total number of processed interest packets by 54.8%, enhance the cache hits of each CR and reduce the number of total hop counts by 51.6%. As a result, the traffic burden of both data packets and interest packets is released and the QoS is ameliorated. Using the two methods of shortcut routing, the overall length of routing paths could be cut down by 28.6–85.7%. Moreover, the length of uploading routing path could be decreased by 8.3–33.3%.

With further research on relevant topics and underlying mechanisms involved in the CJSR scheme, namely, replacement approaches and data-freshness, the CJSR scheme could be partially improved. Possible future works include: (i) implementing and evaluating impacts of various replacement policies on the proposed schemes using similar measure as [60]; (ii) designing an adaptive mechanism that could adjust data packets’ TTL under various circumstances; and (iii) generating experimental evidence of the impacts on the actions caused by modifying TLV values in messages and improving the statistical analysis under heavier traffic pressure.

## Figures and Tables

**Table 1:** Notations.

Notation	Description
CR	Content Router
DC	Data Center
DP	Data Producer
$S_{n}$	A data packet
$I_{n}$	An interest packet for $S_{n}$
*G* = (ξ, *E*)	An undirected graph, ξ and *E* denote vertex and edge respectively
$P_{cache}$	The routing path used for pre-caching
$\xi_{ij}$	An intermediate vertex on the $P_{cache}$
$\xi_{p_{i}}$	A vertex on the $P_{cache}$ adjacent to a Data Producer $P_{i}$.
$\xi_{r_{i}}$	A vertex on the $P_{cache}$ adjacent to a user $r_{i}$
$\xi_{D_{i}}$	A vertex on the $P_{cache}$ adjacent to a Data Center $D_{i}$
$C_{\xi_{i}}$	The storage capacity of $\xi_{i}$
$N_{\xi }$	The number of CRs along the $P_{cache}$
$P_{m}$	The sequence number of a CR in the PathMark element
$A_{\xi_{i,}r_{j}}^{m}$	The Interest frequency of $I_{n}\;$on $\xi_{ij}$ from user $r_{j}$

**Table 2:** Parameters.

Parameter	Values
Routing strategy	Automatic shortest routes
Interest frequency	50 interest packets per second
Cache replacement policy	Least recently used (LRU)
Forwarding strategy	Best-route
Simulation time	10 min
Connection	Point to point
Number of CRs (Scenarios 1–5)	88
Number of CRs (Scenario 6)	113
probability parameter	0.5
cache capacity	250

**Table 3:** Parameters of five scenarios.

Scenario	Number of DPs	Number of Users	Simulation Time	Total Number of Requested Data Packets
Scenario 1	1	1	User 1: 10 min	User 1: 30,000 packets
Scenario 2	1	2	User 1: 10 min User 2: 599 s	User 1: 30,000 packets User 2: 29,950 packets
Scenario 3	1	12	User 1: 10 min Users 2–12: 72 s	User 1: 30,000 packets Users 2–12: 3600 packets
Scenario 4	2	2	User 1: 10 min User 2: 10 min	User 1: 30,000 packets User 2: 30,000 packets
Scenario 5	2	4	Users 1 and 3: 10 min Users 2 and 4: 50 s	Users 1 and 3: 30,000 packets Users 2 and 4: 2500 packets
Scenario 6	6	6	User 1–6: 10 min	Users 1–6: 30,000 packets
